# Plant Extracts and Phytochemicals Targeting *α*-Synuclein Aggregation in Parkinson's Disease Models

**DOI:** 10.3389/fphar.2018.01555

**Published:** 2019-03-19

**Authors:** Hayate Javed, Mohamed Fizur Nagoor Meeran, Sheikh Azimullah, Abdu Adem, Bassem Sadek, Shreesh Kumar Ojha

**Affiliations:** ^1^Department of Anatomy, College of Medicine and Health Sciences, United Arab Emirates University, Al Ain, United Arab Emirates; ^2^Department of Pharmacology and Therapeutics, College of Medicine and Health Sciences, United Arab Emirates University, Al Ain, United Arab Emirates

**Keywords:** α-synuclein, plants, phytochemicals, Parkinson's disease, neuroprotective, natural products, neurotoxicity, bioactive agents

## Abstract

α-Synuclein (α-syn) is a presynaptic protein that regulates the release of neurotransmitters from synaptic vesicles in the brain. α-Syn aggregates, including Lewy bodies, are features of both sporadic and familial forms of Parkinson's disease (PD). These aggregates undergo several key stages of fibrillation, oligomerization, and aggregation. Therapeutic benefits of drugs decline with disease progression and offer only symptomatic treatment. Novel therapeutic strategies are required which can either prevent or delay the progression of the disease. The link between α-syn and the etiopathogenesis and progression of PD are well-established in the literature. Studies indicate that α-syn is an important therapeutic target and inhibition of α-syn aggregation, oligomerization, and fibrillation are an important disease modification strategy. However, recent studies have shown that plant extracts and phytochemicals have neuroprotective effects on α-syn oligomerization and fibrillation by targeting different key stages of its formation. Although many reviews on the antioxidant-mediated, neuroprotective effect of plant extracts and phytochemicals on PD symptoms have been well-highlighted, the antioxidant mechanisms show limited success for translation to clinical studies. The identification of specific plant extracts and phytochemicals that target α-syn aggregation will provide selective molecules to develop new drugs for PD. The present review provides an overview of plant extracts and phytochemicals that target α-syn in PD and summarizes the observed effects and the underlying mechanisms. Furthermore, we provide a synopsis of current experimental models and techniques used to evaluate plant extracts and phytochemicals. Plant extracts and phytochemicals were found to inhibit the aggregation or fibril formation of oligomers. These also appear to direct α-syn oligomer formation into its unstructured form or promote non-toxic pathways and suggested to be valuable drug candidates for PD and related synucleinopathy. Current evidences from *in vitro* studies require confirmation in the *in vivo* studies. Further studies are needed to ascertain their potential effects and safety in preclinical studies for pharmaceutical/nutritional development of these phytochemicals or dietary inclusion of the plant extracts in PD treatment.

## Introduction

Parkinson's disease (PD) is a progressive, debilitating neurodegenerative disease that often begins with the gradual loss of dopaminergic neurons in the substantia nigra pars compacta (SNc) (Herrera et al., 2017). It is a common age-related movement disorder that often appears sporadically (Collier et al., 2017). The pathogenesis of PD remains poorly understood, but emerging evidence implicates various genetic and environmental factors in the initiation and progression of PD (Cannon and Greenamyre, 2013). The multifactorial etiopathogensis of PD includes mitochondrial dysfunction, excitotoxicity, endoplasmic reticulum stress, oxidative/nitrosative stress, and inflammation, along with ubiquitin-proteasome system dysfunction (Moore et al., 2005; Lashuel et al., 2013; Ur Rasheed et al., 2016; Gelders et al., 2018). Altogether, these events lead to the accumulation of abnormal or misfolded α-synuclein (α-syn) protein (Moore et al., 2005; Lashuel et al., 2013; Ur Rasheed et al., 2016; Gelders et al., 2018). Numerous genetic, biochemical, cellular, pathological, and molecular studies indicate PD pathogenesis is associated with environments where α-syn is susceptible to polymerization, aggregation and fibril formation, and propagation (Moore et al., 2005; Hansen and Li, 2012; Lashuel et al., 2013; Gelders et al., 2018; Ghiglieri et al., 2018). The α-syn oligomers cause mitochondrial dysfunction and induce endoplasmic stress, oxidative stress, neuroinflammation, and inhibit proteasomal activity and autophagy (Ghiglieri et al., 2018).

Current PD treatment options, such as dopamine agonists, cholinesterase, and monoamine oxidase inhibitors provide only symptomatic relief (Ellis and Fell, 2017). Dopamine-based drugs have reduced effectiveness in relieving symptoms with disease progression (Ceravolo et al., 2016). The oligomerization and fibrillation of α-syn is linked with the onset and progression of PD (Hansen and Li, 2012), and is believed to be a unique and convincing disease-modification therapeutic strategy for PD, dementia with Lewy body (DLB), and related α-synucleinopathy (Kalia et al., 2015; Török et al., 2016; Brundin et al., 2017). Several molecules including antibodies (Bergström et al., 2016), polyamines (Büttner et al., 2014), heat shock proteins (Cox et al., 2016), chaperones (Friesen et al., 2017), and pharmaceuticals (Lauterbach et al., 2010) have been shown to affect different forms of α-syn (i.e., monomers, soluble oligomers, protofibrils, or fibrils) and oligomerization, fibrillation, and clearance. Therefore, targeting α-syn aggregation, oligomerization, fibrillation, and propagation to reduce α-syn toxicity emerged as an important therapeutic target for slowing or halting disease progression (Kalia et al., 2015; Török et al., 2016; Brundin et al., 2017).

Several recent reviews highlighted the neuroprotective potential of plant extracts and phytochemicals in PD through antioxidant and anti-inflammatory activities (Sarrafchi et al., 2016; da Costa et al., 2017; Mazo et al., 2017; Morgan and Grundmann, 2017; Wang et al., 2017; Zhang et al., 2017; Amro and Srijit, 2018). However, despite the enormous success of antioxidants (whether of synthetic or natural origin) in preclinical studies, coenzyme Q10 (Beal et al., 2014), creatine (Attia et al., 2017), and vitamin E (Ahlskog, 1994) either failed or showed marginal neuroprotection in patients. Recently, α-syn antibodies (PRX002) showed safety in phase 1 studies and were indicated for further phases of clinical studies (Schenk et al., 2017; Jankovic et al., 2018). Similarly, natural products (mainly plant extracts and phytochemicals) emerged to specifically target α-syn (Masuda et al., 2006; Meng et al., 2009, 2010; Caruana et al., 2011; Marchiani et al., 2013). Yet, no comprehensive review is available on these plant extracts and phytochemicals, or on how they target the different steps leading to α-syn oligomerization or fibrillation.

**Figure 1:**
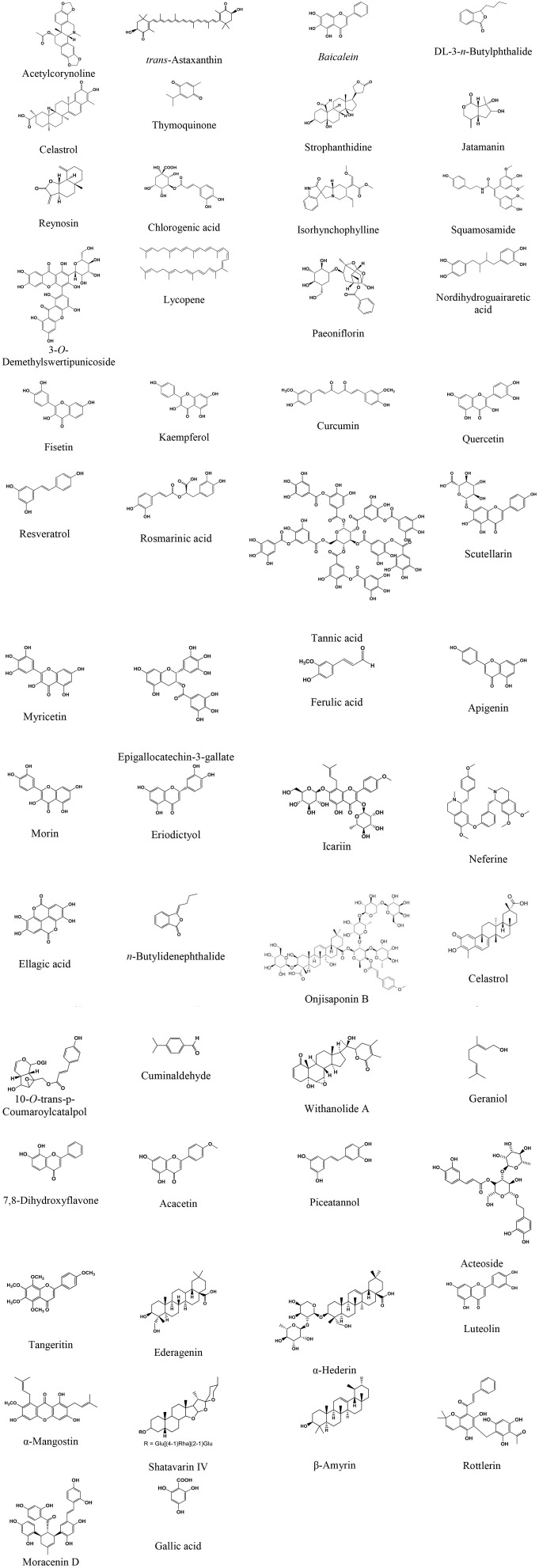
Chemical structure of the phytochemicals attenuating α-synuclein activity in animal models of Parkinson's disease.

**Figure 2:**
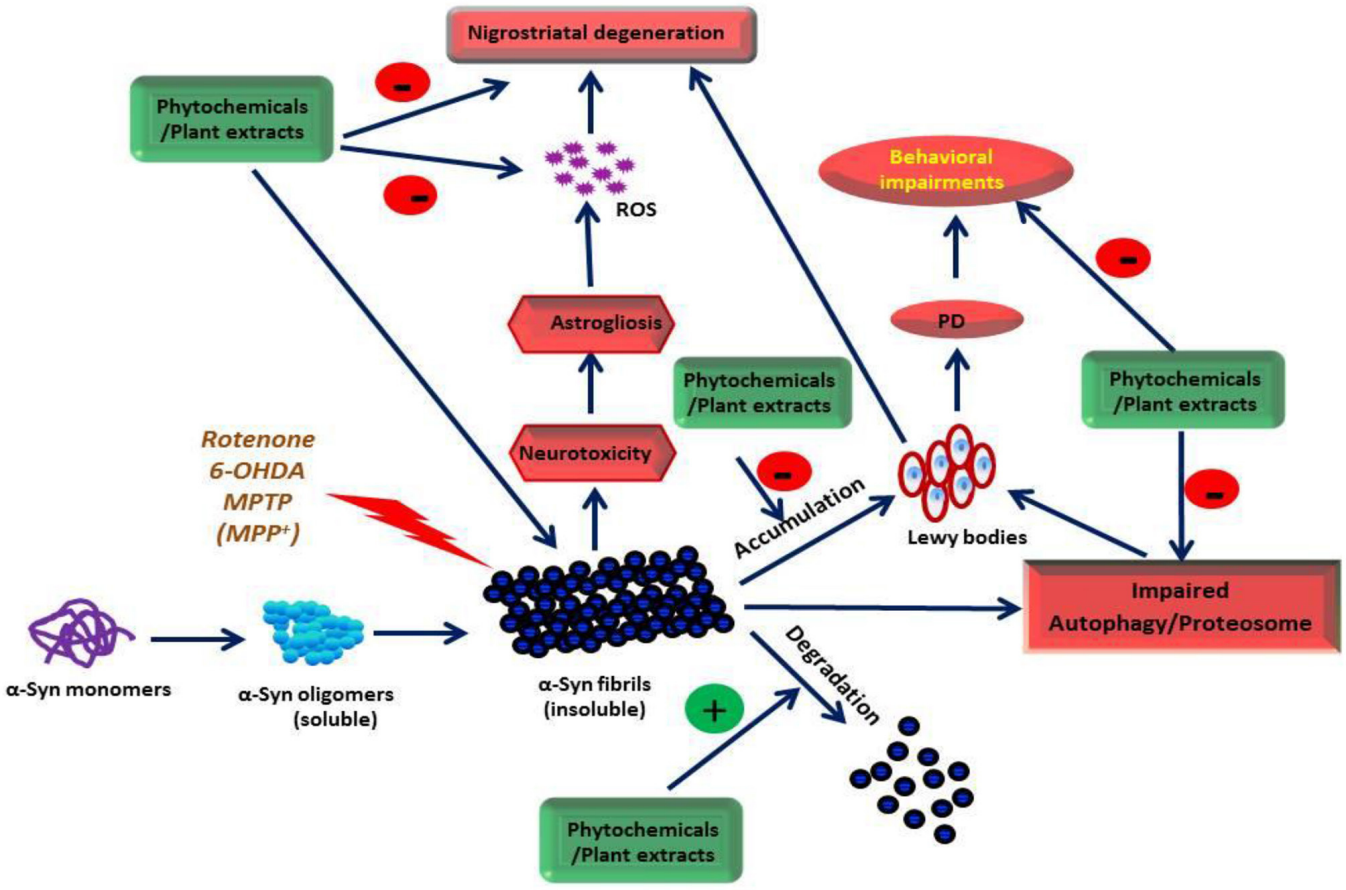
The site of action of the phytochemicals and plant extracts on α-Syn.

This review, therefore, focuses on the neuroprotective properties and mechanism of action of plant extracts, extract-based formulations, and plant-derived phytochemicals that target α-syn oligomerization, fibrillation, aggregation, and toxicity in various experimental PD models. Furthermore, we also elaborate on the suitability of biochemical, biophysical, and neurochemical techniques to evaluate plant extracts and phytochemicals that ameliorate α-syn neurotoxicity. The source of phytochemicals, the models used, and the effect/mechanisms observed are presented in Tables 1–**7**. The chemical structures of these phytochemicals are presented in Figure 1. A scheme on the action of the plant extracts and phytochemicals targeting α-syn is presented in Figure 2.

**Table 1:** The plant extracts and formulations providing neuroprotection in Parkinson's disease models by targeting α-synuclein.

**Plant extract(s) (*Plant name*, family)**	**Experimental model system (s)**	**Effects and mechanisms observed**	**References**
*Acanthopanax senticosus* harms (Siberian Ginseng, Araliaceae)	SH-SY5Y cells overexpressing wild-type or A53T mutant α-syn	■ Inhibits α-syn, caspase-3, Akt, and p-GSK3β ■ Reverses phospho-microtubule-associated tau in cells	Li et al., 2014
*Alaria esculenta* (Winged kelp, Araliaceae)	α-syn aggregation biochemical, biophysical assays	■ Reduces the melting point of α-syn ■ Inhibits aggregation and fibril formation by interacting with an unfolded form of α-syn	Giffin et al., 2017
*Bacopa monnieri* (Waterhyssop, Plantaginaceae)	*Caenorhabditis elegans* expressing human α-syn and 6-OHDA expressing GFP neurons	■ Reduces α-syn aggregation ■ Prevents dopaminergic cell death	Jadiya et al., 2011
*Cinnamon* z*eylanicum* (Cinnamon, Lauraceae)	α-syn aggregation assay and A53T α-syn expression in drosophila	■ Inhibits α-syn aggregation, stabilizes soluble oligomers of α-syn and redirects to “off-pathway” oligomers ■ Improves behavior and cognition	Shaltiel-Karyo et al., 2012
*Centella asiatica* (Asiatic pennywort, Apiaceae)	α-syn aggregation assay	■ Inhibits α-syn aggregation and stabilizes oligomer ■ Disintegrates preformed fibrils	Berrocal et al., 2014
*Carthamus tinctorius* (Safflower extract with flavonoids, Asteraceae)	6-OHDA-induced rat model of PD	■ Improves behavioral performances ■ Reduces α-syn aggregation and astrogliosis ■ Decreases tortuosity and the rate constant of clearance	Ren et al., 2016
*Crocus sativus* L. (Saffron, Iridaceae)	α-syn aggregation, and α-syn fibril dissociation assays	■ Prevents dissociation of fibrils and inhibit α-syn aggregation	Inoue et al., 2018
*Chondrus crispus* (Red seaweed or Irish Moss, Gigartinaceae)	6-OHDA-induced neurodegeneration in transgenic *Caenorhabditis elegans*	■ Reduces α-syn accumulation ■ Attenuates oxidatives stress and improved longevity	Liu et al., 2015
*Corema album* (Portuguese Crowberry, Ericaceae)	Cellular and *in vitro* models of α-syn toxicity and aggregation	■ Promotes non-toxic α-syn and inhibits its aggregation ■ Promotes autophagic flux and reduces oxidative stress	Macedo et al., 2015
*Geum urbanum* (Bennet, colewort, Rosaceae)	α-syn aggregation biochemical, biophysical assays	■ Inhibits α-syn fibrillation dose dependent ■ Disintegrates preformed α-syn fibrils	Lobbens et al., 2016
*Opuntia ficus-indica* (P*rickly pear*, Cactaceae) and *Padina pavonica* (Peacock's tail, brown algae, Dictyotaceae)	PD model of transgenic drosophila expressing human α-syn A53T	■ Increases lifespan and correct behavioral deficit ■ Inhibits fibrillogenesis, stabilize/remodeloligomers	Briffa et al., 2017
*Panax ginseng* (G115) (Asian ginseng, Araliaceae)	β-sitosterol β-d-glucoside-induced PD in rats	■ Prevents dopaminergic loss and locomotor deficits ■ Attenuates α-Syn aggregation, microgliosis, and apoptosis	Van Kampen et al., 2003
*Polygala tenuifolia* (Tenuigenin) (Chinese Senega, Polygalaceae)	SH-SY5Y cells transfected with wild-type or A53T mutant α-syn	■ Improves cell viability ■ Reduces α-syn phosphorylation and PLK3 levels	Zhou et al., 2013
S/B formulation (*Scutellaria baicalensis* Georgi; Baikal skullcap, Lamiaceae and *Bupleurum scorzonerifolfium* Willd)	α-syn aggregation in the infused substantia nigra of rats	■ Attenuates inflammation, apoptosis, oxidative, mitochondrial and ER stress and preserves glutathione ■ Attenuates astrocytosis/microgliosis, improve dopamine ■ Inhibits α-syn aggregation in SNc	Lin et al., 2011
*Rehmannia glutinosa Libosch* (Chinese foxglove, Scrophulariaceae)	Monosodium L-glutamate induced-hippocampal changes in rats	■ Polysaccharides show anxiolytic activity ■ Inhibits down-regulation of β-Syn	Cui et al., 2013
*Scutellaria pinnatifida* (Skullcap, Lamiaceae)	PC12 and primary dopaminergic neurons	■ Dichloromethane and n-butanol extract reduces α-SN aggregation and scavenges free radicals	Sashourpour et al., 2017
Tianma Gouteng Yin (Traditional Chinese medicine decoction)	Rotenone intoxicated and human α-syn transgenic drosophila and SH-SY5Y cells	■ Enhances fly survival and locomotion ■ Reduces the loss of dopaminergic neurons and cytotoxicity ■ Inhibits α-syn and dopaminergic neurons degeneration	Liu et al., 2015

## α-synuclein as a Therapeutic Target for PD

α-syn, a 140-residue presynaptic protein in the brain, plays a key role in the trafficking and fusion of synaptic vesicles and it regulates dopamine release at presynaptic terminals (Burre et al., 2010; Bendor et al., 2013). The physiological concentration of α-syn is 1 μM in the normal human brain and 70 pM in cerebrospinal fluid (Borghi et al., 2000). It exists natively as an unfolded monomer and attains an α-helical secondary structure after binding lipid vesicles. Upon destabilization, this leads to the misfolding and aggregation of α-syn in neurons (Ruipérez et al., 2010; Bartels et al., 2011; Broersen et al., 2018). Monomeric α-syn is an intrinsically disordered protein found in different conformational states. It plays a significant role in many key biochemical processes (Tompa, 2005), as well as in a rising number of diseases involving misfolding, notably PD (Uversky and Dunker, 2010). In dopaminergic neurons, the intracytoplasmic inclusions of α-syn (Spillantini et al., 1998), synphilin-1 (Wakabayashi et al., 2000) and ubiquitin (Kuzuhara et al., 1988) form Lewy bodies, a pathological characteristic of PD. The cascade of α-syn aggregation begins with dimer formation, then tiny oligomers/protofibrils that lead to the development of β-sheet-rich α-syn fibrils. These eventually lead to end-stage fibrils and aggregated α-syn that are the major component of Lewy bodies (Ghiglieri et al., 2018). Thus, in the multistep process of α-syn-mediated neuronal toxicity, oligomerization of α-syn monomers is the primary phase that facilitates the development of intracytoplasmic inclusions and fibrils (Spillantini et al., 1997).

Numerous theories have been proposed on the role of α-syn in initiating dopaminergic neurodegeneration in PD (Herrera et al., 2017; Ghiglieri et al., 2018). These include the interaction of α-syn aggregates with biomolecules, impaired fusion, and trafficking of vesicles, excessive free radical generation, mitochondrial dysfunction, endoplasmic reticulum stress, and synaptic dysfunction (Herrera et al., 2017; Longhena et al., 2017; Ghiglieri et al., 2018). The α-syn protein consists of three distinct domains, where the central region is critical for α-syn fibril aggregation, a key component of Lewy bodies. α-syn can adopt a wide range of conformational structures ranging from compact to fully extended (Winner et al., 2011). The interactions between the N- and C-termini of α-syn play a role in its stabilization into a compact, monomeric conformation that is non-toxic (Bertoncini et al., 2005). The agents that bind to α-syn and form a loop structure between the N- and C-terminus are believed to confer neuroprotection. In contrast, the agents which induce more compressed structures are considered neurotoxic in nature (Karpinar et al., 2009; Lashuel et al., 2013). Mutations in α-syn can contribute to multiple forms of PD including genetic and rare forms of PD with early onset (Singleton et al., 2003; Simon-Sanchez et al., 2009). Monomeric α-syn is a potential therapeutic target as it is an upstream form of the protein during the aggregation process and the etiopathogenesis of PD (Winner et al., 2011; Lashuel et al., 2013; Brundin et al., 2017; Ghiglieri et al., 2018). The agents stabilizing, promoting clearance, degrading misfolded proteins, solubilizing oligomers, or inhibiting the propagation of α-syn aggregates are pharmacologically appropriate and a clinically relevant therapeutic strategy for PD.

## Medicinal Plants Targeting α-synuclein Cascade and Toxicity

Recently, many plant extracts appear to inhibit oligomerization and fibrillization of α-syn, an emerging therapeutic target in PD (Lobbens et al., 2016; Ren et al., 2016; Briffa et al., 2017; Cheon et al., 2017). The plant extracts, which were shown to be neuroprotective in PD, target various pathogenic stages of α-syn conformations ranging from fibrillation to oligomerization in experimental models and are listed in Table 1. Plants, such as *Acanthopanax senticosus [Eleutherococcus senticosus (Rupr. & Maxim.) Maxim.], Bacopa monnieri [Bacopa monnieri (L.) Wettst.], Cinnamon extract precipitate [Cinnamomum verum J. Presl], Centella asiatica [Centella asiatica (L.) Urb.], Panax ginseng [Panax ginseng C.A. Mey.], Polygala tenuifolia [Polygala tenuifolia Willd.], Rehmannia glutinosa [Rehmannia glutinosa (Gaertn.) DC.], Corema album [Trema micranthum (L.) Blume], Opuntia ficus-indica [Opuntia ficus-indica (L.) Mill.], Padina pavonica [Sagina japonica (Sw. ex Steud.) Ohwi], Carthamus tinctorius L*., and *Crocus sativus L*. are neuroprotective in PD by targeting oligomerization, fibrillation, and disaggregation of preformed α-syn fibrils. A scheme is presented in Figure 2 to depict the potential mechanism of action of the plant extracts and phytochemicals on α-syn oligomerization, fibrillation, and aggregation.

Many plant extracts show (often *in vitro*) effects in experimental models of PD by targeting α-syn. However, the bioactive constituents attributing to this effect are not available. *Bacopa monnieri* prevents neurodegeneration in A53T α-syn-induced PD in *Caenorhabditis elegans* (Jadiya et al., 2011). However, the chemical constituents collectively known as bacosides have not been investigated in experimental PD models or their effect on α-syn. *Centella asiatica (L.) Urb.*, known as Asiatic pennywort, reportedly prevents α-syn aggregation *in vitro* (Berrocal et al., 2014). Yet, the principal constituent asiatic acid failed to prevent α-syn aggregation. Meanwhile, asiaticoside and madecassic acid have not been investigated for their effects on α-syn. Cinnamon extract precipitate reportedly inhibits α-syn aggregation and stabilizes oligomers *in vitro* and *in vivo* in A53T α-syn-induced PD in flies (Shaltiel-Karyo et al., 2012). However, cinnamaldehyde, a major ingredient of cinnamon extract has not yet been investigated. *Eucalyptus citriodora* improves climbing ability and attenuates oxidative stress in transgenic drosophila expressing human α-syn (Siddique et al., 2013a). The effects of the bioactive contents citronellol, linalool, and isopulegol of *Eucalyptus citriodora* on α-syn are not known. *Crocus sativus* L., popularly known as saffron, is widely used for its color, flavor, and aroma in food and beverages. Saffron and its constituents, such as crocin-1, crocin-2, crocetin, safranal, and the crocetin structural analogs hexadecanedioic acid, norbixin, and trans-muconic acid, were found to affect α-syn fibrillation and aggregation (Inoue et al., 2018). However, some crocetin analogs failed to affect α-syn aggregation and dissociation. *Sorbus alnifolia*, also known as Korean mountain ash, improved viability of rat pheochromocytoma (PC12) cells while also improving the longevity, food sensing, and reducing dopaminergic neurodegeneration in *Caenorhabditis elegans* model of PD (Cheon et al., 2017). However, the extract failed to alter α-syn aggregation in the NL5901 strain (Cheon et al., 2017).

From the perspective of traditional medicine, targeting α-syn with plant extracts containing phytochemicals could be considered beneficial using dietary intervention. This could be due to the synergy in action and superior therapeutic effects, along with polypharmacological properties (Wagner and Ulrich-Merzenich, 2009; Wu et al., 2013). The fraction that termed active from *Radix Polygalae* was found more potent than the constituent, where onjisaponin B increased mutant huntingtin removal and reduced α-syn aggregation. This plant could be a good source of phytochemicals and a template for novel small molecule inhibitors of α-syn (Wu et al., 2013). Plant-based formulations, such as S/B which contain extracts of *Scutellaria baicalensis Georgi* and *Bupleurum scorzonerifolfium* and a traditional Chinese medicine decoction known as *Tianma Gouteng Yin*, were also found to diminish α-syn accumulation and aggregation in experimental PD models (Lin et al., 2011).

The majority of plant extracts used in traditional medicines are based on long-established knowledge of their health benefits, time tested safety due to ancient use, and potential therapeutic effects. However, some plants are not as beneficial as documented or are detrimental; the essential oil from *Myrtus communis*, which is popular in the Zoroastrian community for aroma (Morshedi and Nasouti, 2016), increases α-syn fibrillation and enhances α-syn toxicity in human neuroblastoma cells (Morshedi and Nasouti, 2016). This study suggests that essential oils used in aromatherapy should be investigated for their potential neurotoxicity or neurodegenerative ability.

The attenuation of α-syn toxicity by plant extracts validates traditional claims of medicinal plants. It may also provide the basis for dietary or nutritional inclusion of these plants in foods to achieve neuroprotective effects. This is not only based on antioxidant approaches but also inhibition of α-syn aggregation. However, in-depth studies are needed for a dietary or therapeutic recommendation on the use of plant extracts in humans.

## Plant Extracts and Phytochemicals as Pharmacological Chaperones for PD

Pharmacological chaperoning is emerging as a potential therapeutic approach for the treatment of numerous diseases associated with single gene mutations (Srinivasan et al., 2014). These chaperones are small molecules that bind proteins and stabilize them against proteolytic degradation or protect them from thermal denaturation. Furthermore, they assist in or prevent certain protein-protein assemblies similar to the molecular chaperones (Ringe and Petsko, 2009). Chaperoning is beneficial in cystic fibrosis (Chanoux and Rubenstein, 2012), Gaucher's disease (Sawkar et al., 2002), nephrogenic diabetes insipidus (Tamarappoo and Verkman, 1998), and retinitis pigmentosa (Noorwez et al., 2003). Mechanistically, ligand-mediated chaperoning is believed to correct receptor mislocalization and inhibit mutant proteins from forming toxic intracellular aggregates (Loo and Clarke, 2007). This has been shown to be successful with the pharmacological chaperone, tafamadis, in a clinical trial for the treatment of transthyretin familial amyloid polyneuropathy (Coelho et al., 2013). Several of the molecular chaperones, such as Hsp70, Hsp40, and torsin A either prevent the misfolding of proteins or promote the degradation and elimination of misfolded proteins; they provide a novel therapeutic approach in PD (Dimant et al., 2012).

Although molecular chaperoning is therapeutically significant in α-syn-associated neurodegeneration, the structural heterogeneity and deficiency of persistent structural components for α-syn creates a major issue in the discovery, design, and development of small molecules targeting α-syn (Lester et al., 2009). Plant-derived phytochaperones are a good source of molecules that target protein misfolding in neurotherapeutics (Bernd, 2008). In a chaperone-based approach, *Ginkgo biloba* is being utilized to search for lead molecules in drug discovery and in the development of protein-misfolding diseases leading to neurodegeneration (Kastenholz and Garfin, 2009). Thus, plant extracts and phytochemicals are a novel source of pharmacological chaperones for a disease-modifying approach that could be promising against neurodegenerative diseases. Following the reductionist approach of drug discovery from plant extracts, it is also important to characterize the bioactive constituents contributing to these pharmacological effects.

## Phytochemicals Targeting α-synuclein Assembly and Toxicity

The phytochemicals are non-nutritive secondary metabolites that are heavily utilized for drug discovery and development; they remain an important source of drugs (Beutler, 2009; Henrich and Beutler, 2013). The phytochemicals that target α-syn at different stages of pathogenicity are represented in Table 2 (*in vitro* studies), Table 3 (*in vivo* studies), and Table 4 (*in vitro* and *in vivo*, both studies), respectively. A benefit of the phytochemicals is their huge structural diversity that offers lead structures for drug discovery and development. They belong to many classes, such as alkaloids, saponins, carotenoids, lignans, glycosides, etc. Briefly, the alkaloids are a nitrogen-containing, structurally-diverse group of secondary metabolites that are protective against neurodegenerative diseases (Hussain et al., 2018). To name a few, galantamine is used in the pharmacotherapy of mild to moderate Alzheimer's disease. Many of the alkaloids, such as acetylcorynoline, 3α-acetoxyeudesma-1,4 (15),11 (13)-trien-12, 6α-olide, corynoxine B, dl-3-n-butylphthalide, isorhynchophylline, and squamosamide attenuate neurotoxicity in experimental models by directly inhibiting α-syn aggregation or fibril formation.

**Table 2:** The phytochemicals targeting α-synuclein in the *in vitro* models of Parkinson's disease.

**Phytochemicals (*Plant name*, family)**	***In vitro* model system**	**Effects and mechanisms observed**	**References**
3α-Acetoxyeudesma-1,4(15),11(13)-trien-12,6α-olide *(Laurus nobilis*, Lauraceae*)*	Dopamine-induction and α-syn formation in neuroblastoma cells (SH-SY5Y)	■ Inhibits apoptosis by decreasing ofcaspase-3 and p53 activation and increasing Bcl-2 ■ Suppresses tyrosinase activity and ROS generation ■ Suppresses quinoprotein and α-syn formation	Koo et al., 2011
Alpinin A and B (Diarylheptanoid from *Alpinia officinarum*, Zingiberaceae)	α-syn aggregation assay	■ Inhibits α-syn aggregation, respectively	Fu et al., 2017
*Baicalein* *(Scutellaria baicalensis*, Lamiaceae)	α-syn aggregation assay	■ Inhibits the formation of α-syn fibrils ■ Disaggregates α-syn fibrils involving Tyr	Zhu et al., 2004
Baicalein (Flavonoid from *Scutellaria baicalensis*, Lamiaceae)	Dopaminergic cell lines (SN4741) overexpressing wild-type α-syn or A53T mutant type α-syn	■ Inhibits α-syn fibrillation by binding covalently ■ Promotes degradation of α-syn fibrils and polymerization to reduce its propagation and transmission ■ Enhances cell viability and increased macroautophagy	Li et al., 2017
dl-3-n-Butylphthalide (*Apium graveolens*, Apiaceae)	MPP^+^-induced cellular injury in PC12 cells	■ Reduces cytotoxicity and α-syn accumulation ■ Suppresses oxidative stress and mitochondrial permeability ■ Upregulates LC3-II and its colocalization with α-syn	Huang et al., 2010
Celastrol (*Tripterygium wilfordii* Celastraceae)	Rotenone-induced cell death in SH-SY5Y cells	■ Alleviates oxidative stress and protects from cell death ■ Activates autophagy and increases LC3-II/LC3 I ratio ■ Enhances α-syn clearance	Deng et al., 2013
Chlorogenic acid (*Coffee Arabica*, Rubiaceae)	α-syn-induced toxicity in PC12 cells	■ Inhibits oxidation of dopamine and its interaction with α-syn ■ Inhibits α-syn oligomerization, cytotoxicity, and apoptosis	Teraoka et al., 2012
Costunolide (*Laurus nobilis*, Lauraceae)	Human dopaminergic SH-SY5Y cells	■ Regulates dopamine metabolism-associated genes ■ Decreases α-syn levels and apoptosis	Ham et al., 2012a
Curcumin (*Curcuma longa*, Zingiberaceae)	α-syn aggregation assay and α-syn induced cytotoxicity in SH-SY5Y cells and induced A53T α-syn PC12 in cells	■ Increases α-syn solubility and prevents oligomerization ■ Attenuates apoptosis, ROS, and mitochondrial depolarization ■ Reduces formation, aggregation, and accumulation of α-syn ■ Downregulates mTOR/p70S6K signaling and recovers suppressed macroautophagy ■ Binds to preformed oligomers/fibrils, alter the hydrophobic surface ■ Binds specifically to oligomer intermediates and reduces numbers	Ono and Yamada, 2006; Pandey et al., 2008; Wang et al., 2010; Liu et al., 2011; Gadad et al., 2012; Jiang et al., 2013; Singh et al., 2013
Curcumin-glucoside	α-syn aggregation biochemical assay	■ Prevents oligomer and fibrilformation ■ Improves binding with oligomers and enhances α-syn solubility and prevents fibrillation of α-syn ■ Solubilizes oligomers by disintegrating preformed fibrils	Gadad et al., 2012
Curcumin derivatives: Dehydrozingerone, O-methyl, zingerone, biphenyl analogs	α-syn aggregation biochemical assay and PC12 cells model of PD	■ Biphenyl analogs of dehydrozingerone and O-methyl-dehydrozingerone inhibit α-syn aggregation process ■ Displays the best antioxidant properties	Marchiani et al., 2013
Curcumin pyrazole and curcumin isoxazole	α-syn aggregation biochemical, biophysical and cell based assays	■ Curcumin pyrazole and derivative exhibit better potency ■ Arrests fibrillization and disrupting preformed fibrils ■ Prevents A11 conformation in protein that imparts toxicity ■ Decreases fast aggregating A53T mutant form of α-syn	Ahsan et al., 2015
Curcumin	α-syn in genetic synucleinopathy mouse line overexpresses wild-type α-syn	■ Improves gait impairments ■ Increases phosphorylated α-syn in presynaptic terminals without affecting α-syn aggregation	Spinelli et al., 2015
Curcumin with β-cyclodextrin	α-syn aggregation assay	■ Inhibits aggregation and ■ Brakes up preformed aggregates, exhibit synergy in their action at low concentrations	Gautam et al., 2014
Curcumin, myricetin, rosmarinic acid, nordihydroguaiaretic acid, and ferulic acid	Biophysical assays for α-syn and electrophysiological assays for long-term potentiation in mouse hippocampal slices	■ Inhibits α-syn oligomerization and structure conversion ■ Directly bound to the N-terminal region of α-syn ■ Ameliorates α-syn aggregation and α-syn synaptic toxicity ■ Prevents process, reducing the neurotoxicity of αS oligomers ■ Ameliorates α-syn synaptic toxicity in long-term potentiation	Takahashi et al., 2015
Crocin-1,2, safranal and crocetin, and its analogs; hexadecanedioic acid, norbixin, and trans-muconic acid (*Crocus sativus* L., °Iridaceae)	α-syn aggregation and fibril dissociation assays	■ Prevent dissociation of fibrils and inhibit α-syn aggregation ■ Crocetin appears most potent and thereafter norbixin ■ Other analogs of crocetin fail to affect α-syn aggregation and dissociation	Inoue et al., 2018
(-)-Epigallocatechingallate (EGCG)	Oligomerization, fibrillization, and preformed fibrils of α-syn using biophysical techniques	■ Inhibits α-syn aggregation concentration dependently ■ Decreases fibrillar size and toxicity of oligomeric/fibrillar aggregates of α-syn	Jha et al., 2017
(-)-Epigallocatechingallate (EGCG)	Fe^+3^-induced fibrillation of α-syn in transduced-PC12 cells	■ Inhibits ROS and β-sheet-enriched α-syn fibrils by chelating Fe(III)	Zhao et al., 2017
(-)-Epigallocatechin-3-gallate (EGCG)	α-syn aggregation biochemical and biophysical assays	■ Influences aggregate toxicity, morphology, seeding competence, stability, and conformational changes ■ Affects aggregation kinetics, oligomeric aggregation, binds to cross-beta sheet aggregation intermediates	Andrich and Bieschke, 2015
(-)-Epi-gallocatechine gallate (EGCG)	Fibril formation in incubates; SNCA fluorophore α-syn-HiLyte488 binding to plated SNCA and α-syn-HiLyte488 binding to aggregated SNCA in post-mortem PD tissue	■ Concentration-dependent inhibition of α-syn aggregation ■ ED_50_ of EGCG inhibition of α-syn-HiLyte488 was 250 nM ■ Blocks concentration dependently α-syn-HiLyte488 ■ Binds to SNCA by instable hydrophobic interactions ■ Appear remodeling agent of SNCA aggregates and a disease modifying agent for PD	Xu et al., 2016
3-*O-*demethyl swertipunicoside (*Swertia punicea*, Gentianaceae)	MPP^+^-induced neurotoxicity in PC12 cells	■ Alleviates oxidative stress by regulating SOD, MDA, and ROS ■ Down-regulates Bax and involve a caspases-mediated pathway ■ Inhibits AIF translocation and α-syn aggregation	Zhou et al., 2013
Fistein (*Polyphenolic compound)*	MPTP/MPP^+^-induced neurotoxicity in PC12 cells	■ Decreases cytotoxicity, apoptosis, and inflammation ■ Decreases α-syn expression	Patel et al., 2012
Flavonoids (*48 polyphenolic compounds)*	α-syn aggregation assay	■ Inhibits α-syn fibrillation and disaggregates preformed fibrils	Meng et al., 2009
Gallic acid (*Flavonoid of reference)*	Thioflavin T fluorescence assays and transmission electron microscopy imaging, ion mobility-mass spectrometry	■ Inhibits the formation of α-syn mediated amyloid fibrils ■ Interacts with α-syn transiently ■ Stabilizes its native structure	Liu et al., 2014
Ginsenosides (Rb1) (*Panax ginseng*, Araliaceae)	α-syn aggregation and toxicity using biophysical, biochemical and cell-culture techniques	■ Inhibits α-syn fibrillation and disaggregate preformed fibrils and inhibit the seeded polymerization of α-syn ■ Stabilizes soluble non-toxic oligomers with no β-sheet content	Ardah et al., 2015
Isorhynchophylline (*Uncaria rhynchophylla Miq.*, Rubiaceae)	Neuronal cell lines, including N2a, SH-SY5Y, and PC12 cells, and primary cortical neurons	■ Clears α-syn oligomers and α-syn/synphilin-1 aggresomes ■ Activates autophagy-lysosome pathway independent of the mTOR pathway rather dependent on the function of Beclin 1 ■ Decreases α-syn levels in dopaminergic neurons	Lu et al., 2012
Jatamanin11 (*Valeriana jatamansi*, Caprifoliaceae)	*In silico* analysis using Homo sapiens α-syn gi|49456267 from NCBI database	■ Shows good interaction α-syn in homology modeling	Bagchi and Hopper, 2011
Kaempferol (*a polyphenolic compound)*	α-syn aggregation biochemical assay	■ Inhibits the formation of α-syn ■ Destabilizes preformed α-syn	Ono and Yamada, 2006
Luteolin (*Dietary flavonoid)*	Arsenite-induced apoptosis in the dopaminergic PC12 cells	■ Scavenges ROS production, and promotes apoptosis ■ Reduces α-syn aggregation	Wu et al., 2017
α-Mangostin (*Garcinia mangostana L.*, Guttiferae)	*In vitro* model of Parkinson's disease induced by rotenone in SH-SY5Y cells	■ Reduces α-syn aggregation and TH loss ■ Reduces reactive oxygen species and caspases 3 and 8 ■ Restores mitochondrial membrane potential and cellular ATP	Hao et al., 2017
Moracenin D (*Morus alba*, Moraceae)	Dopamine-induction in neuroblastoma, SH-SY5Y cells	■ Upregulates nurr1 levels and down-regulate α-syn levels	Ham et al., 2012b
Neferine (*Lotus seed embryo of Nelumbo nucifera*, Nelumbonaceae)	GFP-LC3 autophagy detection platform in PC-12 cells with mutant toxic proteins, including huntingtin or α-syn	■ Induces autophagy through an AMPK-mTOR pathway ■ Reduces expression and toxicity of mutant huntingtin by autophagy-related gene 7 (Atg7) dependent mechanism	Wong et al., 2015
Onjisaponin B (Triterpenoid saponin from *Radix Polygalae*, Polygalaceae)	Mutant α-syn in PC-12 cells	■ Accelerates clearance of mutant A53T α-syn ■ Induces autophagy via the AMPK-mTOR signaling pathway ■ Reduces oligomerization of α-syn	Wu et al., 2013
Oxidized quercetin (Chalcantrione, benzyfuranone, quercetinchinone)	α-syn aggregation biochemical assay	■ Inhibits fibrillation of α-syn ■ Disaggregates α-syn fibrils ■ Inhibits fibrillation and stabilizes oligomers	Zhu et al., 2013
Polyphenols with β-cyclodextrin (Baicalein, curcumin, EGCG, and resveratrol)	α-syn aggregation in mouse neuroblastoma cell lines (N2a cells)	■ Inhibited α-syn aggregation and disaggregate fibrils ■ CURCUMIN appears most efficient followed by baicalein, EGCG, and resveratrol	Gautam et al., 2017
Piceatannol, ampelopsin A and isohopeaphenol (Stilbene compounds)	α-syn aggregation biochemical and biophysical assays in PC12 cells	■ Protects against α-syn-induced membrane damage ■ Rescues against α-syn-induced toxicity ■ Inhibits α-syn fibril formation and destabilizes preformed	Temsamani et al., 2016
Paeoniflorin (*Paeoniae alba*, Paeoniaceae)	MPP^+^/acidosis-induced cytotoxicity in PC12 cells expressing α-syn	■ Upregulates LC3-II expression showing autophagy ■ Reduces MPP^+^ cytotoxicity and α-syn accumulation ■ Enhances autophagic degradation of α-syn	Sun et al., 2011
Quercetin, (-)-Epigallocatechin gallate (EGCG) and cyanidin-3-glucoside (C3G)	Primary cortical neuron cultures exposed to oxidative insult	■ EGCG crosses blood brain barrier faster, then C3G ■ EGCG and C3G reduces necrosis and apoptosis by 30–40% ■ Quercetin, EGCG, and C3G inhibited α-syn fibrillation ■ EGCG appears most promising neuroprotective compound	Pogacnik et al., 2016
Rottlerin (Polyphenol from berry fruits or kamala tree, *Mallotus Philippinensis*, Euphorbiaceae)	α-syn aggregation biochemical assay	■ Prevents aggregation of numerous amyloid precursors (α-syn, amyloid-β, prion proteins, and lysozyme)	Maioli et al., 2012
Resveratrol (*Red grapes*, Vitaceae)	Rotenone-treated human SH-SY5Y cells and wild-type α-syn, A30P, or A53T α-syn expressing PC12 cells	■ Protects against apoptosis and enhanced degradation of α-syn ■ Shows AMPK-SIRT1-mediated autophagy induction ■ Activates SIRT1 and prevents α-syn aggregation	Albani et al., 2009; Wu et al., 2011
3,6-bis-O-di-O-galloyl-1,2,4-tri-O-galloyl-β-d-glucose (Tannin from *Rhus typhina*, Anacardiaceae)	α-syn aggregation biochemical and biophysical assays	■ Interacts very strongly with human serum albumin through a “sphere of action” mechanism ■ Time-dependent inhibition of α-synuclein aggregation	Sekowski et al., 2017
Strophanthidine (*Strophanthus Kombe & gratus*, Apocynaceae)	SNCA 5′UTR driven luciferase expression	■ Blocks *SNCA* expression (~1 μM IC_50_) in neural cells	Rogers et al., 2011
Theaflavins (TF1, TF2a, TF2b, and TF3) (*Camelia sinensis*, Theaceae)	α-syn aggregation biochemical assay	■ Stimulates α-syn assembly into non-toxic, spherical aggregates	Grelle et al., 2011
Thymoquinone (*Nigella sativa*, Ranunculaceae)	α-syn-induced synaptic toxicity in rat hippocampal cells and human induced pluripotent stem cell (iPSC)-derived neurons	■ Reduces the α-syn-induced loss of synaptophysin ■ Enhances synaptic vesicles recycling in the presence of α-syn ■ Protects iPSC-derived neurons and maintain firing activity ■ Protects against mutated β-SynP123H-induced synaptic activity	Alhebshi et al., 2014

**Table 3:** The phytochemicals showed neuroprotective effects in the *in vivo* models of Parkinson's disease by targeting α-synuclein.

**Phytochemicals** ***(Plant name*, family*)***	***In vivo* animal model**	**Effects and mechanisms observed**	**References**
Apigenin *(Flavone found in fruits and vegetables)*	Unilateral stereotaxic intranigral infusion of ROT-induced PD in rats	■ Improves behavioral, biochemical and mitochondrial enzymes ■ Attenuates pro-inflammatory cytokines release and NF-κB expression ■ Inhibits neurotrophic factors and α-syn aggregation ■ Enhances TH and dopamine D2 receptor expression	Anusha et al., 2017
Acteoside (*Cistanche deserticola* or *Cistanche tubulosa*, Orobanchaceae)	Rotenone-induced PD in rats	■ Inhibits α-syn, caspase-3 activity and microtubule-associated protein 2 (MAP2) downregulation ■ Binds and inhibits caspase-3 *in silico* and showed neuroprotection	Yuan et al., 2016
Acacetin (O-methylated flavone from Asteraceae)	Caenorhabditis elegans model system	■ Improves lifespan, survival, stress resistance ■ Enhances antioxidant and stress resistance genes ■ Inhibits α-syn aggregation and age pigment lipofuscin	Asthana et al., 2016
Acetylcorynoline *(Corydalis bungeana* *Turcz*, Papaveraceae*)*	Transgenic *C. elegans* (OW13) expressing human α-syn, GFP in dopaminergic neurons and 6-OHDA-induced PD	■ Decreases 6-OHDA-induced DA neuron degeneration ■ Prevents α-syn aggregation and recovers lipid content ■ Restores food-sensing behavior in 6-OHDA-treated animals ■ Suppresses apoptosis by decreasing egl-1 expression ■ Increases rpn5 expression that enhances the activity of proteasomes	Fu et al., 2014a
Apocyanin (*Picrorhiza kurroa* Royle ex Benth, Plantains)	Lipolysaccharide-injection in substantia niagra-induced PD in rats	■ Ameliorates proinflammatory cytokines, improves behavior ■ Inhibits NADPH oxidase, caspase 3, 9 and TUNEL positivity ■ Inhibits α-syn deposition and prevents dopaminergic neurons	Sharma et al., 2016
Acetylcorynoline (*Corydalis bungeana*, Papaveraceae)	*Caenorhabditis elegans* strain (BZ555) expresses the green fluorescent protein in dopaminergic neurons, and a transgenic strain (OW13) express h α-syn in muscle cells PD model	■ Appears safe and devoid of adverse effect in animals ■ Decreases dopaminergic degeneration in BZ555 strain ■ Prevents α-syn aggregation and recovers lipid contents ■ Restores food-sensing behavior, and dopamine levels ■ Prolongs life-span in 6-OH-treated N2 strain ■ Decreases egl-1 expression to suppress apoptosis pathways ■ Increases rpn5 expression to enhance proteasomes activity	Fu et al., 2014a
Baicalein (Flavonoid from *Scutellaria baicalensis*, Lamiaceae)	Intranigral infusion of MPP^+^ in rat brain	■ Attenuates α-syn aggregation ■ Inhibits inflammasome activation and cathepsin B production ■ Inhibits apoptosis (caspases 9 and 12, and autophagy (LC3-II)	Hung et al., 2016
*n*-Butylidenephthalide (*Angelica sinensis*, Apiaceae)	*Caenorhabditis elegans* express green fluorescent protein in neurons, BZ555 and a transgenic expresses human α-syn (OW13)	■ Attenuates dopaminergic degeneration and prolongs life-span ■ Reduces α-syn accumulation ■ Restores dopamine, lipid content and food-sensing behavior ■ Blocks *egl-1* expression that inhibits apoptosis ■ Enhances *rpn-6* expression to increase proteasomes activity	Fu et al., 2014b
Curcumin (*Curcuma longa*, Zingiberaceae)	Interaction of curcumin and α-syn in genetic synucleinopathy of α-syn-GFP mouse line overexpresses α-syn	■ Chronic and acute curcumin treatment improves gait impairments and increases phosphorylated forms of α-syn at cortical presynaptic terminals in α-syn-GFP line ■ Increases phosphorylated α-syn in terminals without affecting α-syn aggregation	Spinelli et al., 2015
Alginate-curcumin nanocomposite	Supplemented with diet to *Drosophila melangoster*	■ Delays climbing disability in flies ■ Reduces oxidative stress and apoptosis in the brain of PD flies	Siddique et al., 2013b
α-Linolenic acid	*Caenorhabditis elegans* wild type N2 and transgenic (UA44) exposed to 6-OHDA	■ Improves locomotion, pharyngeal pumping, and lifespan ■ Shows a visibly significant reduction in neuronal degeneration ■ Increases GFP expression within in neurons	Shashikumar et al., 2015
Squamosamide (N-[2-(4-Hydroxy-phenyl)-ethyl]-2-(2,5-dimethoxy-phenyl)-3-(3-methoxy-4-hydroxy-phenyl) acrylamide) *(Annona glabra*, Annonaceae)	6-OHDA-induced PD in rats	■ Improves motor dysfunction and behavior ■ Enhances dopamine level and TH activity ■ Decreases α-syn expression mediated by the Akt/mTOR pathway ■ Reduces RTP801 expression, a protein in the pathogenesis of PD	Bao et al., 2012
Geraniol *(Monoterpene from rose oil, palmarosa oil, and citronella oil)*	MPTP-induced PD in C57BL/6 mice	■ Reduces α-syn aggregation in dose dependent manner ■ Improves nigral dopamine, TH and dopamineric terminals in striatum ■ Improves neuromuscular disability and Lewy body aggregation	Rekha et al., 2013
Irisflorentin (*Belamcanda chinensis* L. DC., Iridaceae)	Transgenic or 6-hydroxydopamine-induced PD in *Caenorhabditis elegans*	■ Prevents α-syn accumulation ■ Improves dopaminergic neurons, food-sensing, and life-span ■ Promotes rpn-3 expression to enhance the activity of proteasomes ■ Down-regulates egl-1 expression to block apoptosis pathways	Chen et al., 2015a
Lycopene *(Red grapes, peanuts)*	Rotenone-induced PD in mouse	■ Increases the TH content and decreases α-syn and LC3-B positive neurons	Liu et al., 2013
N-2-(4-hydroxy-phenyl)-ethyl]-2-(2, 5-dimethoxy-phenyl)-3-(3-methoxy-4-hydroxy-phenyl)-acrylamide) (FLZ, a novel synthetic derivative of squamosamide from a Chinese herb)	Chronic PD mouse model induced by MPTP combined with probenecid (MPTP/p) and subacute PD models	■ Improves motor behavior and dopaminergic neuronal function ■ Elevates dopaminergic neurons, dopamine level, and TH activity ■ Decreases α-syn phosphorylation, nitration, and aggregation ■ Decreases interaction between α-syn and TH, which eventually improved dopaminergic neuronal function ■ Activates Akt/mTOR phosphorylation signaling pathway	Bao et al., 2015
Salidroside (Phenylpropanoid glycoside from *Rhodiola rosea* L., Crassulaceae)	MPTP/MPP(+) models of Parkinson's disease and 6-OHDA and overexpresssion of WT/A30P-α-syn in SH-SY5Y cells.	■ Protects dopaminergic neurons and regulates apoptotic proteins caspase-3,6 and 9, cyt-c and Smac release and Bcl-2/Bax ■ Reduces α-syn aggregation ■ Protects cells and cell viability mainly through recovering the 20S proteasome activity ■ Decreases pSer129-α-syn and promotes the clearance of α-syn	Wang et al., 2015b, Li et al., 2018
Shatavarin IV (Steroidal glucosides, syn: asparinin B in roots of *Asparagus racemosus*, Asparagaceae)	*Caenorhabditis elegans* model of PD	■ Improves antioxidant and stress defense genes ■ Raises dopamine levels, inhibits lipids ■ Inhibits α-syn aggregation involving ubiquitin proteasomal system	Smita et al., 2017
2,3,5,4′-tetrahydroxy stilbene-2-O-β-D-glucoside *(Polygonum multiflori*, Polygonaceae)	APPV717I transgenic mice expressing α-syn in the hippocampus	■ Prevents α-syn overexpression at an early and late stage in the hippocampus ■ Inhibits production of dimer and tetramer of α-syn protein ■ Reverses the increased expression of α-syn	Zhang et al., 2013
2,3,5,4′-tetrahydroxystilbene-2-O-β-D-glucoside *(Polygonum multiflori*, Polygonaceae)	Memory and movement functions and its mechanisms related to synapses and α-syn in aged mice	■ Inhibits α-syn aggregation and α-syn levels in the hippocampus ■ Improves memory, movement and protects synaptic ultrastructure ■ Enhances synaptophysin, phosphorylated synapsin I and post-synaptic density protein 95 (PSD95) and calcium/calmodulin-dependent protein kinase II (p-CaMKII) expression	Shen et al., 2015
10-O-trans-p-Coumaroylcatalpol *(Premna integrifolia syn: Premna serratifolia*, Verbenaceae*)*	Transgenic *Caenorhabditis elegans* model of PD expressing α-syn	■ Inhibits α-syn aggregation ■ Extends life span, stress resistance and reduces oxidative stress ■ Enhances longevity promoting transcription factors	Shukla et al., 2012
Withanolide A (Steroidal lactone from *Withania somnifera* L. Dunal, Solanaceae)	Transgenic *Drosophila melanogaster* model	■ Improves lifespan and delays age-associated physiological changes ■ Inhibits α-syn aggregation and modulation of acetylcholine.	Akhoon et al., 2016

**Table 4:** The phytochemicals showed neuroprotective effects in both, the *in vitro* and *in vivo* models of Parkinson's disease by targeting α-synuclein.

**Phytochemicals (*Plant name*, family)**	***In vitro* and *in vivo* models**	**Effects and observed mechanisms**	**References**
Astaxanthin (3,3′-dihydroxy-β, β′-carotene-4, 4′-dione)	MPTP/MPP^+^-induced PD in mouse and neuroblastoma cells (SH-SY5Y)	■ Inhibits apoptosis regulating Bax, Bcl-2 and caspase-3 expression ■ Reduces α-syn and argyrophilic neurons ■ Increases TH+ve neurons and antioxidant activity	Lee et al., 2011
2-Cyano-3, 12-dioxooleana-1,9-dien-28-oic acid (*a derivative of oleanolic acid*)	MPTP-induced PD in mice and 3-NP-neurotoxicity in mice and SH-SY5Y cells	■ Reduces oxidative/nitrosative stress and activate the Nrf2/ARE pathway ■ Preserves dopaminergic neurons, reduced α-syn accumulation	Yang et al., 2009
Corynoxine B (*Uncaria rhynchophylla Miq.*, Rubiaceae*)*	Neuronal cell lines and N2a and SHSY-5Y cells and drosophila model of PD	■ Promotes autophagosomes formation in fly fat bodies ■ Enhances clearance of wild-type and A53T α-syn ■ Induces autophagy by Akt/mTOR pathway	Chen et al., 2014
(-)-Epicatechin gallate (EGCG) (*Camelia sinensis*, Theaceae)	α-syn aggregation biochemical assays, A53T α-syn expressing SH-SY5Y cells, transgenic drosophila model expressing normal human α-syn	■ Inhibits α-syn fibrillogenesis and disaggregates large, mature α-syn fibrils into smaller, amorphous protein aggregates and α-syn tandem repeat in the aggregation ■ Blocks genomic responses and accumulation of α-syn in SNc ■ Forms a new type of unstructured, non-toxic α-syn ■ Shows a dose-dependent delay in the loss of climbing ability ■ Reduces oxidative stress and apoptosis in the brain ■ Remodels α-syn amyloid fibrils into disordered oligomers ■ Inhibits preformed oligomers to permeabilize vesicles, induce cytotoxicity in cells and immobilizes C-terminal region and reduces binding of oligomers to membranes ■ Does not affect oligomer size distribution or secondary structure ■ Reduces membrane affinity of the oligomer to prevent cytotoxicity	Mandel et al., 2004; Ehrnhoefer et al., 2008; Bae et al., 2010; Bieschke et al., 2010; Ma et al., 2010; Yoshida et al., 2013; Lorenzen et al., 2014; Siddique et al., 2014
Eicosanoyl-5-hydroxytryptamide (*Coffee Arabica*, Lamiaceae)	MPTP-model of PD in mice and cultured primary microglia/astrocytes and MPP-induced PD model of SH-SY5Y cells	■ Prevents oxidative stress, cytotoxicity, and neuroinflammation ■ Preserves dopaminergic neurons and improves neuronal integrity ■ Reduces JNK activation, striatal dopamine, and TH content ■ Ameliorates MPP^+^-demethylation of phosphoprotein phosphatase 2A, the key of the cellular phosphoregulatory network ■ Ameliorates protein aggregation and phosphorylation	Lee et al., 2013
Ellagic acid	Cell-based and cell-independent *in vitro* showing nitrosative stress mediated S-nitrosylation (SNO), the SNO-PDI formation is linked to the aggregation of α-syn and α-syn:synphilin-1 deposits in the PD brain	■ Scavenges NOx radicals and protect cells from SNO-PDI formation via rotenone insult both, cell-based and cell-independent *in vitro* ■ Mitigates nitrosative-stress-induced aggregation of synphilin-1 but also α-syn and α-syn: synphilin-1 composites (Lewy-like neurites) in PC12 cells ■ Lowers rotenone-instigated reactive oxygen species and reactive nitrogen species in PC12 cells ■ Inhibits apoptosis and interferes with SNO-PDI formation	Kabiraj et al., 2014
Nordihydroguaiaretic acid (*Larrea tridentata*, Zygophyllaceae)	Drosophila expressing human α-syn and α-syn aggregation biochemical assay	■ Delays loss of climbing ability of flies ■ Inhibits the formation of α-syn ■ Destabilizes preformed α-syn	Ono and Yamada, 2006; Caruana et al., 2012; Siddique et al., 2012
Reynosin *(Laurus nobilis*, Lauraceae*)*	DA-induced PD model in SH-SY5Y cells and 6-OHDA induced PD in rats	■ Reverse E6-associated protein, α-syn levels ■ Appears more potent than apomorphine	Ham et al., 2013
Tanshinone I & IIA (*Salvia miltiorrhiza*, Lamiaceae)	Transgenic *Caenorhabditis elegans* PD model (NL5901) and *in vitro*	■ Disaggregates fibrils, the transformation of α-syn from unstructured coils to β-sheets and reduce oligomer/fibril formation ■ Inhibits α-syn aggregation and alleviates aggregated α-syn induced membrane disruption and extends life span	Ji et al., 2016
Tea polyphenols (flavanol-related catechins in black/green tea)	MPTP-induced PD models in mouse and monkey and cultured dopaminergic cells	■ Alleviates motor impairments and dopaminergic injury in monkeysinhibits α-syn oligomers in cultured cells, striatum, brain reduces intracellular α-syn oligomers in neurons treated with α-syn oligomers, MPTP and increases cell viability	Chen et al., 2015b
Trehalose (natural sugar in fungi and plants)	Autophagy-induction in NB69 cells and mice model of Lewy body disease	■ Induces autophagy and increases autophagosomes ■ Increases autophagic and chaperon molecules in mice brain ■ Suppressesinsoluble α-syn and apoptosis	Tanji et al., 2015

Saponins are an abundant group of secondary metabolites that can be classified as triterpenoids, steroids, and glycosides (Dinda et al., 2010). Their effects in neurodegenerative, neuropsychiatric, and affective disorders were recently reviewed (Sun et al., 2015). Saponins possess surface-active and amphipathic properties (Lorenzen et al., 2014) that may contribute to their membrane-permeabilizing actions and surfactant-based disruption of α-syn fibril formation. Many of the glucosides, such as 3-O-demethylswertipunicoside, jatamanin 11, paeoniflorin, 2,3,5,4′-tetrahydroxy stilbene-2-O-β-D-glucoside, 10-O-trans-p-coumaroylcatalpol and strophanthidine attenuate neurotoxicity in experimental models by directly inhibiting α-syn aggregation or fibril formation. Similarly, many terpenoids, such as celastrol, 2-cyano-3, 12-dioxo-oleana-1,9-dien-28-oic acid, geraniol, reynosin, thymoquinone, and ginkgolide A, B, and C attenuate neurotoxicity in experimental models by directly inhibiting α-syn aggregation or fibril formation. However, asiatic acid failed to prevent α-syn aggregation and protofibril formation (Masuda et al., 2006).

Dietary intake of polyphenolic compounds is protective against neurodegeneration as evidenced from many epidemiologic and experimental studies (Ho and Pasinetti, 2010; Caruana et al., 2016; da Costa et al., 2017). Popular polyphenols in food are curcumin (present in turmeric), oleuropein (present in olive oil), resveratrol (present in grapes), catechins (present in black and green tea), astaxanthin (a carotenoid present in vegetables and fruits) and lycopene (present in tomato) (da Costa et al., 2017). Polyphenols inhibit α-syn aggregation and fibrillation (Masuda et al., 2006; Caruana et al., 2011, 2012; Sivanesam and Andersen, 2016) and formation of amyloid protofilaments and fibrils (Kumar et al., 2012; Velander et al., 2017) and confer protective effects in neurodegenerative diseases. Masuda et al. (2006) tested 79 compounds from different chemical classes of compounds including polyphenols, benzothiazoles, terpenoids, steroids, porphyrins, lignans, phenothiazines, polyene macrolides, and Congo red and its derivatives for their potential to inhibit α-syn assembly. Out of 39 polyphenolic compounds tested, 26 were found to inhibit α-syn assembly. These findings establish that polyphenols constitute a major class of compounds that can inhibit the assembly of α-syn. Several of them inhibited α-syn filament assembly with IC50 values in the low micromolar range. Caruana et al. (2011) investigated 14 polyphenolic compounds and black tea extract containing theaflavins and found that baicalein, scutellarein, myricetin, (-)-epigallocatechin-3-gallate (EGCG), nordihydroguaiaretic acid and black tea extract are the ideal candidates to investigate in experimental models for their direct effect on the inhibition of α-syn oligomer formation. The polyphenolic compounds are believed to interact with receptors or plasma membrane transporters and activate intracellular signaling pathways. Among several polyphenols, EGCG associates with the laminin receptor on vascular cells (Tachibana et al., 2004). Currently, numerous polyphenolic compounds have been studied for their effect on α-syn aggregation, fibrillation, elongation, nitration, and oligomerization using biophysical and biochemical techniques (Meng et al., 2010; Caruana et al., 2011, 2012; Takahashi et al., 2015). The list of these compounds is presented in Table 5. A scheme is presented in Figure 2 to depict the mechanism of action of the plant extracts and phytochemicals on α-syn oligomerization, fibrillation, and aggregation. An overview of some important phytochemicals which target α-syn aggregation and fibrillation and appear ideal candidates for further development is presented below.

**Table 5:** The polyphenol compounds investigated for their action on α-synuclein fibrillation, aggregation, and cytotoxicity.

**Polyphenolic compounds**	**References**
Apigenin, baicalein, (-)-catechin, (-)-catechin gallate, chlorogenic acid, curcumin, cyaniding, daidzein, delphinidin, 2,2′-dihydroxybenzophenone, 4,4′-dihydroxybenzophenone, dopamine chloride, (-)-epicatechin, (-)-epicatechin 3-gallate, epigallocatechin, epigallocatechin gallate, exifone, (-)-gallocatechin, (-)-gallocatechin gallate, gingerol, gossypetin, hinokiflavone, hypericin, kaempferol, luteolin, myricetin, naringenin, 2,3,4,2′,4′-pentahydroxybenzophenone, procyanidin B1, procyanidin B2, Purpurogallin, quercetin, rosmarinic acid, rutin, (+)-taxifolin, 2,2′,4,4′-tetrahydroxybenzophenone, theaflavine, (+)-R-tocopherol, 2,3,4-trihydroxybenzophenone (39 polyphenols)	Masuda et al., 2006
Quercetin (3,5,7,3′,4′-Pentahydroxyflavone dehydrate), Fisetin (3,7,3′,4′-Tetrahydroxyflavone), T-601 (3′,4′-Dihydroxyflavonol), 22-344 (3,6,3′,4′-Tetrahydroxyflavone), 22-318 (3,6,2′,3′-Tetrahydroxyflavone), G-500/Gossypetin (3,5,7,8,3′,4′-Hexahydroxyflavone), C-101/Myricetin (3,5,7,3′,4′,5′-Hexahydroxyflavone), Rutin (Quercetin-3-rutinoside), K-102/Kaempferol (3,5,7,4′-Tetrahydroxyflavone), 020065/Isorhamnetin (3′-Methoxy-3,5,7,4′-Tetrahydroxyflavone), 020067/Galangin (3,5,7-Trihydroxyflavone), 021140S/Tamarixetin (4′-Methoxy-3,5,7,3′-Tetrahydroflavone), 22-324 (6,2′,3′-Trihydroxyflavone), D-406 (2′,3′-Dihydroxyflavone), D-258 (3′,4′-Dihydroxyflavone), D-116 (5,6-Dihydroxy-7-Methoxyflavone), 22-357 (5,6-Dihydroxyflavone), 22-336 (6,7,3′-Trihydroxyflavone), D-112 (6,7-Dihydroxyflavone), Luteolin (5,7,3′,4′-Tetrahydroxyflavone), 22-340/Tricetin (5,7,3′,4′,5′-Pentahydroxyflavone), 22-341 (7,3′,4′,5′-Tetrahydroxyflavone), 021165/6-HP (5,6,7,4′-Tetrahydroxyflavone), B-101/Baicalein (5,6,7-Trihydroxyflavone), 22-323 (6,2′,3′-Trimethoxyflavone), D-407 (2′,4′-Dihydroxyflavone), 021104S/Chrysoeriol (4′,5,7-Trihydroxy-3′-Methoxyflavone0, 021108S/Diosmetin (5,7,3′-Trihydroxy-4′-Methoxyflavone), Wogonin (5,7-Dihydroxy-8-Methoxyflavone), H-114 (3′-Hydroxy-5,6,7,4′-Tetramethoxyflavone), Epigallo Catechin Gallate (EGCG) [(2R,3R)-2-(3,4,5-Trihydroxyphenyl)-3,4-dihydro-1(2H)-benzopyran-3,5,7-triol 3-(3,4,5-trihydroxybenzoate)], Epicatechin gallate (ECG) [(2R,3R)-2-(3,4-Dihydroxyphenyl)-3,4-dihydro-1(2H)-benzopyran-3,5,7-triol 3-(3,4,5-trihydroxybenzoate)], 020976S/Catechin [(+)-3,3′4′,5,7-Flavanepentol (2H)-benzopyran-3,5,7-triol/(2R,3R)-2-(3,4-Dihydroxyphenyl)-3,4-dihydro-1], T-116 (6,7,4′-Trihydroxyisoflavone), T-415 (7,3′,4′-Trihydroxyisoflavone), 19-612 (3′,4′-Dimethoxy-7-hydroxyisoflavone), D-101/Daidzein (7,4″-Dihydroxyisoflavone), F-103/Formononetin (7-Hydroxy-4′-methoxyisoflavone), Biochanin A (5,7-Dihydroxy-4′-methoxyisoflavone), 020056/Eriodictyol (2-[3,4-Dihydroxyphenyl] 2,3-dihydro-5,7-dihydroxy-4H-1benzopyran-4-one), H-103/Hesperetin (5,7,3′-Trihydroxy-4′-methoxyflavanone), 020091/Homoeriodictyol (5,7,4′-Trihydroxy-5′-methoxyflavanone), Hesperidin/Hesperetin-7-O-rutinoside, 020411/Alizarin (1,2-Dihydroxyanthraquinone), Chrysophanol (1,8-Dihydroxy-3-methylanthraquinone), Emodin (1,3,8-Trihydroxy-6-methylanthraquinone), D-105/Fustin (3,7,3′,4′-Tetrahydroxyflavone), 021037 (3,3′,4′,5′,7-Pentahydroxyflavanone) (48 polyphenols)	Meng et al., 2010
Apigenin, baicalein, epigallocatechin gallate, genistein, ginkgolide B, morin, myricetin (Myr), nordihydroguaiaretic acid, purpurogallin trimethyl ether, quercetin, resveratrol, rosmarinic acid, scutellarein, tannic acid, theaflavins (14 polyphenols)	Caruana et al., 2011
Apigenin, baicalein, EGCG, genistein, ginkgolide B, morin, nordihydroguaiaretic acid, propyl gallate, purpurogallin trimethyl ether, resveratrol, scutellarein, and black tea extract (BTE; >80% theaflavins) (12 polyphenoilc compunds)	Caruana et al., 2012
Benzoic acid and derivatives, such as 2-Hydroxybenzoic acid (salicylic acid), 3-Hydroxybenzoic acid, 4-Hydroxybenzoic acid, 2,3-dihydroxybenzoic acid, 2,4-dihydroxybenzoic acid, 2,5-dihydroxybenzoic acid (gentisic acid), 2,6-dihydroxybenzoic acid, 3,4-dihydroxybenzoic acid, 3,5-dihydroxybenzoic acid, 2,4,6-trihydroxybenzoic acid, 3,4,5-trihydroxybenzoic acid (gallic acid), 3,4,5-trihfluorobenzoic acid, 3,4,5-trimethoxybenzoic acid, 4-methoxybenzoic acid (Benzoic acid derivatives)	Ardah et al., 2014
Myricetin, curcumin, rosmarinic acid, nordihydroguaiaretic acid, and ferulic acid	Takahashi et al., 2015
Curcumin, baicalein, (-)-epigallocatechin gallate, and resveratrol	Gautam et al., 2017

### Baicalein

Baicalein is a flavone isolated from the roots of *Scutellaria baicalensis* Georgi (“Huang Qin” in Chinese), a reputed plant in traditional Chinese medicine (Gasiorowski et al., 2011) and Scutellaria *pinnatifida* grown in Iran (Sashourpour et al., 2017). In many studies, baicalein was shown to prevent α-syn oligomerization and fibrillation (Bomhoff et al., 2006; Meng et al., 2009; Caruana et al., 2011; Gasiorowski et al., 2011; Sashourpour et al., 2017). Baicalein interacts with α-syn through a tyrosine residue. Following oxidation, it generates quinone metabolites that bind covalently with a lysine side chain in α-syn. It prevents fibril formation and degrades preformed fibrils at low micromolar concentrations (Zhu et al., 2004). In another study, its non-covalent binding with α-syn and covalent modification by the oxidized form restricts the conformational changes in the unfolded protein that results in α-syn monomer and oligomer stabilization (Meng et al., 2009). The oligomers cause impairment of neuronal membrane integrity that results in disruption or permeabilization of the membrane, impairment of calcium homeostasis, and cell death (Caruana et al., 2012).

Baicalein prevents α-syn fibrillation and protects against neurotoxicity by preventing α-syn oligomer formation in SH-SY5Y and HeLa cells (Lu et al., 2011). It also stabilizes the oligomers, prevents further fibrillation (Hong et al., 2008) and tandem repeats of α-syn in the aggregation process (Bae et al., 2010). Further, it prevents the formation of annular protofibrils of α-syn induced by copper and reduces the β-sheet contents (Zhang et al., 2015). In another study, using JC-1, a probe that binds the α-syn C-terminal region, baicalein differentiated the α-syn fibrillation states (monomeric, oligomeric intermediate, and fibrillar forms) and reconfirmed the defibrillation action of baicalein on α-syn (Lee et al., 2009). In PC12 cells, it ameliorates cytotoxicity, mitochondrial depolarization, and inhibits proteasome inhibition induced by E46K, an α-syn point mutation that mimics familial PD (Jiang et al., 2010). In a recent study, baicalein induces autophagy, increases cell viability and reduces α-syn in the media of dopaminergic cell lines (SN4741) overexpressing A53T-syn (Li et al., 2017). Baicalein diminished the transmission of α-syn and prompted the polymerization of α-syn to a big complex rather than promoting clearance (Li et al., 2017). A recent study in rotenone-induced PD in rats showed reduced α-syn oligomer formation along with behavioral improvement and neurotransmitters in the striatum. However, it failed to reduce α-syn mRNA expression but prevented the transition from α-syn monomers to oligomers (Hu et al., 2016). Furthermore, baicalein attenuated α-syn aggregate formation, induced autophagy, inhibited apoptosis, reduced inflammation, and restored dopamine in PD induced by MPP^+^ infusion in the SNc of mice (Hung et al., 2016).

The baicalein derivative N′-benzylidene-benzohydrazide also attenuated oligomer formation (Kostka et al., 2008). Baicalein in combination with β-cyclodextrin (β-CD) synergistically inhibited α-syn aggregation and disaggregated preformed fibrils even at very low concentrations (Gautam et al., 2017). A combination of baicalein with specific proteolytic peptide sequences of α-syn was developed for targeted drug delivery and found to prevent α-syn fibrillation (Yoshida et al., 2013). Integrating evidence from *in vitro* and *in vivo* studies, baicalein appears to be a potential drug to inhibit α-syn aggregation, fibrillation, and propagation among the neurons.

### Curcumin

Curcumin, chemically known as diferuloylmethane, is one of the most popular natural leads to drug discovery and development from turmeric (Molino et al., 2016). It is reputed for its dietary importance and health benefits and is the most studied phytochemical in experimental and clinical studies (Molino et al., 2016). It is a beneficial treatment in neurodegenerative diseases, including PD, and has antioxidant, anti-inflammatory, and antiapoptotic properties (Kim et al., 2012; Singh et al., 2013; Ji and Shen, 2014). Ono and Yamada (2006) found that curcumin possesses anti-fibrillogenic activity by inhibiting α-syn fibril formation and destabilizing preformed fibrils (Ono and Yamada, 2006). It was found to inhibit oligomerization of mutant α-syn into higher molecular weight aggregates (Pandey et al., 2008) and induce the dissociation of α-syn fibrils (Shoval et al., 2008). Curcumin treatment on mesencephalic cells did not affect α-syn fibril formation but enhanced LRRK2 mRNA and protein expression in rats (Ortiz-Ortiz et al., 2010). In neuroblastoma cells, curcumin attenuates cytotoxicity from aggregated α-syn, ROS generation, and diminished caspase-3 activation (Wang et al., 2010). In PC12 cells, curcumin ameliorates A53T mutant α-syn-induced PD (Liu et al., 2011). Further, curcumin reduces mutant α-syn accumulation by restoring macroautophagy, a process in the degradation pathway that clears proteins in cells by activating the mTOR/p70S6K signaling pathway (Jiang et al., 2013). Mechanistically, curcumin preferentially binds oligomeric intermediates rather than monomeric α-syn (Singh et al., 2013). Also, it binds strongly to the hydrophobic non-amyloid-β component of α-syn (Ahmad and Lapidus, 2012). The ordered structure is vital for effective binding and affects the extent of binding and potential in inhibiting oligomers or fibrils (Singh et al., 2013). The conformational and reconfiguration changes appear to govern the binding of curcumin to α-syn (Ahmad and Lapidus, 2012; Tavassoly et al., 2014). Curcumin, in combination with β-cyclodextrin, showed a synergistic inhibition of α-syn aggregation and degraded the preformed aggregates into monomers at very low concentrations (Gautam et al., 2014, 2017). Gautam et al. (2017) further demonstrated that a balanced arrangement of the phenolic groups, benzene rings, and flexibility attributes to the ability of curcumin. The phenolic groups enhance curcumin interactions with α-syn monomers as well as oligomers. In PC12 cells transfected with recombinant plasmids, α-syn-pEGFP-A53T downregulated α-syn expression or oligomer formation by regulating apoptosis-mediated mitochondrial membrane potential (Chen et al., 2015a). The effect of curcumin on α-syn observed *in vitro* was reconfirmed *in vivo* in genetic mouse models of synucleinopathy (Spinelli et al., 2015). Curcumin increased phosphorylated forms of α-syn at cortical presynaptic terminals but had no direct effect on α-syn aggregation. However, curcumin improved motor and behavioral performance (Spinelli et al., 2015).

Curcumin is less stable and soluble and has limited oral bioavailability. To improve its stability, solubility, and oral bioavailability, many nanoformulations or structural analogs have been developed (Gadad et al., 2012; Kundu et al., 2016; Taebnia et al., 2016). Curc-gluc, a modified curcumin preparation, inhibits α-syn oligomerization and fibrillation (Gadad et al., 2012). In another study, dehydrozingerone, zingerone; an O-methyl derivative of dehydrozingerone and their biphenyl analogs were investigated for their cytoprotective effects in PC12 cells challenged with H_2_O_2_, MPP^+^, and MnCl_2_ (Marchiani et al., 2013). The biphenyl analogs of dehydrozingerone and O-methyl-dehydrozingerone prevent α-syn aggregation; the biphenyl zingerone analog is the most potent inhibitor and has the most potent antioxidant activity. This activity was attributed to the hydroxylated biphenyl scaffold in the pharmacophore (Marchiani et al., 2013). In another study, stable curcumin analogs, such as curcumin pyrazole, curcumin isoxazole, and their derivatives, were evaluated against α-syn aggregation, fibrillation, and toxicity. Curcumin pyrazole and its derivative N-(3-Nitrophenyl pyrazole) curcumin reduces A53T-α-syn-induced neurotoxicity by preventing fibrillation and disrupting preformed fibrils (Ahsan et al., 2015). Taebnia et al. (2016) developed amine-functionalized mesoporous silica nanoparticles of curcumin to enhance its bioavailability and evaluated its effect against cytotoxicity and α-syn fibrillation (Taebnia et al., 2016). This nanoformulation showed interaction with α-syn species and prevented fibrillation with negligible effect on cytotoxicity (Taebnia et al., 2016). A nanoformulation containing curcumin and piperine with glyceryl monooleate nanoparticles coated with various surfactants was developed for targeted delivery to enhance its bioavailability in the brain (Kundu et al., 2016). The nanoformulation has been shown to attenuate oxidative stress, apoptosis, prevent α-syn oligomerization and fibrillation, and induce autophagy. Another nanoformulation prepared with lactoferrin by sol-oil chemistry ameliorates rotenone-induced neurotoxicity in dopaminergic SK-N-SH cells (Bollimpelli et al., 2016). This nanoformulation exhibited better availability, improved cell viability, attenuated oxidative stress, and reduced tyrosine hydroxylase and α-syn expression. Nine curcumin analogs were synthesized by substitution of groups on the aromatic ring which alters the hydrophobicity, promotes stability, and facilitates binding with the fibrils as well as oligomers (Jha et al., 2016). Some of the analogs showed improved stability and appeared to interact with oligomers and preformed fibrils. The analogs exhibited differential binding patterns and augmented α-syn aggregation, generating different kinds of amyloid fibrils. The liposomal nanohybrid of curcumin with polysorbate 80-modified cerasome was developed for targeted drug delivery in the striatum and showed better half-life and bioavailability (Zhang et al., 2018). This nanoformulation ameliorated motor deficits and improved dopamine and tyrosine hydroxylase expression by promoting α-syn clearance in a mouse model of MPTP-induced PD (Zhang et al., 2018). Curcumin inhibited α-syn aggregates in dopaminergic neurons and attenuated oxidative stress, inflammation, apoptosis, and motor deficits in a rat model of lipopolysaccharide-induced PD (Sharma and Nehru, 2018). These reports demonstrate the effect of curcumin on α-syn aggregation- and fibrillation-induced neurotoxicity but further studies are still needed to demonstrate therapeutic success.

### Cuminaldehyde

Cuminaldehyde is isolated from many edible plants including *Artemisia salsoloides, Aegle marmelos*, and spices cumin *(Cuminum cyminum L.)* and is used as a food additive and flavoring agent in many cuisines in the Middle East, South Asia, and Mediterranean countries. Cuminaldehyde isolated from Iranian cumin showed to inhibit α-syn fibrillation (Morshedi et al., 2015). It prevented α-syn fibrillation even in the presence of seeds with negligible disaggregating effect on the preformed fibrils of α-syn. Interestingly, it was found to be superior to baicalein, a known inhibitor of α-syn fibrillation and blocked protein assembly into β-structural fibrils that were attributed to interaction with amine and aldehyde groups in the chemical structure (Morshedi and Nasouti, 2016).

### Catechins, Theaflavins, and (-)-Epigallocatechin-3-Gallate (EGCG)

Catechins, the polyphenolic compounds present in black and green tea, are protective in neurodegenerative diseases (Caruana and Vassallo, 2015; Jha et al., 2017; Xu et al., 2017; Pervin et al., 2018). Theaflavins present in fermented black tea inhibits fibrillogenesis of α-syn and amyloid-β formation (Grelle et al., 2011). These compounds facilitate the assembly of amyloid-β and α-syn into non-toxic, spherical aggregates which are unable to undergo seeding to form amyloid plaques. They were also found to remodel the formed amyloid-β fibrils into non-toxic aggregates and these effects were comparable to EGCG. Theaflavins also appeared less vulnerable to oxidation in air and exhibited better activity in oxidizing environments in comparison with EGCG (Grelle et al., 2011).

One of the most popular catechins, (-)-Epigallocatechin 3-gallate (EGCG), is a flavonol compound predominantly present in green tea, a popular beverage across the world. EGCG inhibited α-syn aggregation and fibrillation in a concentration-dependent manner (Šneideris et al., 2015; Xu et al., 2017), and by disaggregating mature and large α-syn fibrils into smaller, non-toxic, amorphous aggregates (Ehrnhoefer et al., 2008). EGCG binds directly to the natively unfolded polypeptides and inhibits their conversion into toxic intermediates (Ehrnhoefer et al., 2008). It induces a conformational change without their disassembly into monomers or small diffusible oligomers (Bieschke et al., 2010). It appears to bind directly with β-sheet-rich aggregates and reduces its concentration required to induce conformational changes (Liu et al., 2018). Furthermore, it showed neuroprotection against free radicals and α-syn toxicity by chelating Fe (III) in PC12 cells transfected with α-syn and exposed to β-sheet-enriched α-syn fibrils (Zhao et al., 2017). EGCG appears to disaggregate α-syn fibrils by preventing the amyloid formation of α-syn tandem repeat and destabilizing α-syn fibrils into soluble amorphous aggregates (Bae et al., 2010). This study also revealed that the tandem repeat of α-syn may be used as a molecular model to study the mechanism of α-syn aggregation (Bae et al., 2010). Further, EGCG also prevents α-syn aggregation and accumulation by activating the hypoxia-inducible factor (HIF)-1 signaling mechanism that controls α-syn aggregation by regulating antioxidant and iron homeostasis (Weinreb et al., 2013).

Lorenzen et al. (2014) showed that EGCG has potential to prevent α-syn oligomer formation and attenuate the oligomer cytotoxicity by preventing vesicle permeabilization and blocking the membrane affinity of syn to bind and immobilize in the C-terminal region. Though, it failed to affect the oligomer size distribution or secondary structure. Recently, in primary cortical neuron cultures challenged with oxidative injury, quercetin, EGCG, and cyanidin-3-glucoside inhibited fibrillation of α-syn and apoptosis (Pogacnik et al., 2016). Further, it decreased amyloid fibril formation on the surface of liposomal membranes and generates compact oligomers following off-pathway, as well as facilitating the conversion of active oligomers into amyloid fibrils (Yang et al., 2017). A combination of EGCG with specific α-syn proteolytic peptide sequences was developed for targeted drug delivery and found to prevent the α-syn fibrillation (Yoshida et al., 2013). In combination, this evidence suggests EGCG could be a promising treatment in neurodegenerative diseases and a good candidate for pharmaceutical development and dietary inclusion.

### Gallic Acid

Gallic acid, a type of phenolic acid chemically known as 3,4,5-trihydroxybenzoic acid, is found in free form or as part of the hydrolyzable tannins in many plants, such as gallnuts, sumac, witch hazel, tea leaves, and oak bark (Kosuru et al., 2018). Gallic acid and esters are well-known food additives, nutritional supplements, and a common reagent in the pharmaceutical analysis (Kosuru et al., 2018). Over the last few decades, many investigators showed the antioxidative, antiapoptotic, cardioprotective, neuroprotective and anticancer properties of gallic acid and gallates (Blainski et al., 2013; Choubey et al., 2018; Kosuru et al., 2018). It is used as a reference compound for the quantification of the phenolic contents in biochemical assays; Folin-Ciocalteau assay or Folin's phenol reagent or Folin-Denis reagent which determines the antioxidant power in gallic acid equivalents (Blainski et al., 2013). The polyphenolic compound gallic acid and its structurally similar benzoic acid derivatives elicit anti-aggregating effects (Ardah et al., 2014). Gallic acid impedes α-syn fibrillation and disaggregates the preformed fibrils of α-syn in a battery of biophysical, biochemical, and cell viability assays. In addition to inhibiting aggregation and disaggregation, it also binds to soluble and non-toxic oligomers devoid of β-sheet content and confers structural stability. Numerous benzoic acid derivatives have been developed using structure-activity relationship and all inhibit α-syn fibrillation (Ardah et al., 2014). The number of hydroxyl groups and their presence on the phenyl ring in these structural derivatives of gallic acid are believed to attribute to the potential mechanism in binding and inhibiting α-syn fibrillation. Furthermore, gallic acid prevents α-syn amyloid fibril formation, stabilizes the extended intrinsic structure of α-syn, and reacts rapidly in biochemical assays (Liu et al., 2014).

### Ginsenosides

Ginseng, also known as red ginseng (*Panax ginseng*, Araliaceae), is a popular source of saponins and is reputed in the folk medicine of the Far East countries. It has shown neuroprotective effects in numerous neurodegenerative diseases including PD (Van Kampen et al., 2003; Chen et al., 2005; Luo et al., 2011). The ginseng extract, abbreviated as G115, confers neuroprotection against MPTP and its neurotoxic metabolite, MPP^+^ in murine models of PD (Van Kampen et al., 2003). Van Kampen et al. (2014) reported that G115 treatment reduces dopaminergic cell loss, microgliosis, the buildup of α-syn aggregates, and improves locomotor activity and coordination in rats chronically exposed to the dietary phytosterol glucoside, β-sitosterol β-d-glucoside, which recapitulates features of PD.

Several studies identified the active constituents of ginseng known as ginsenosides, a group of 60 compounds that possess a wide range of pharmacological and physiological actions (Mohanan et al., 2018; Zheng et al., 2018). Several ginsenosides, Rg1, Rg3, and Rb1, were investigated for their effect on α-syn aggregation using biophysical and biochemical techniques (Ardah et al., 2015; Heng et al., 2016). Upon oral treatment, Rg1 attenuated neurodegeneration in a mouse model of MPTP-induced PD by inhibiting pro-inflammatory cytokines; reducing mortality, behavioral defects, and dopamine neuron loss; and correcting ultrastructure changes in the SNc (Heng et al., 2016). Rg1 also reduced oligomeric, phosphorylated, and disease-related α-syn in the SNc. In contrast, a separate study identified only Rb1 as a strong inhibitor of α-syn fibrillation; it also disaggregated preformed fibrils and inhibited the seeded polymerization of α-syn (Ardah et al., 2015). Further, Rb1 binds to the oligomers and causes stabilization of soluble, non-toxic oligomers with negligible involvement of β-sheets that depicts a novel mechanism of action (Ardah et al., 2015). Although the authors did not find a significant effect of Rg1 and Rg3 on α-syn aggregation in cellular models (Ardah et al., 2015). In one of the study, Rg3, another ginsenoside present in *Panax ginseng*, reduced α-syn expression in stress models (Xu et al., 2013). The evidence of Rg3-mediated changes in α-syn in stress models needs to be investigated in PD (Xu et al., 2013). A detailed investigation is required to understand the observed differences in the *in vitro* and *in vivo* studies.

### Resveratrol

Resveratrol, a natural phytoestrogen found in grapes and red wine, is reputed for its neuroprotective properties by attenuating oxidative stress, mitochondrial impairment, inducing apoptotic cell death and promoting autophagy (Caruana et al., 2016; Ur Rasheed et al., 2016). Wu et al. (2011) showed that resveratrol enhanced α-syn degradation in PC12 cells expressing α-syn by activating autophagy and mediating the induction of AMP-activated protein kinase (AMPK) mammalian silent information regulator 2 (SIRT1) signaling mechanism. This reduces protein levels of microtubule-associated protein 1 light chain 3 (LC3-II) and preserves neuronal cells. AMPK is a serine/threonine kinase which acts as a metabolic energy sensor to maintain energy balance; upon activation, it induces neuronal cell apoptosis and decreases SIRT1 leading to activation of the ubiquitin-proteasome pathway by enhancing ubiquitination and promoting SUMOylation that may be important in reducing the progression of neurodegeneration (Wu et al., 2011). The induction of autophagy and apoptotic pathways represents an important approach in the therapeutic targeting of α-syn (Ghavami et al., 2014). Further, in MPTP-induced PD in mice, resveratrol corrected the behavioral and motor deficits and attenuated neurodegeneration by inducing autophagy of α-syn via activation of SIRT1 and subsequent deacetylation of LC3 (Guo et al., 2016). Recently, in an effort to enhance the bioavailability to attain therapeutic benefits, resveratrol was prepared with β-CD; this combination was found synergistic in showing activity at very low concentrations to prevent α-syn aggregation as well as disaggregate preformed fibrils (Gautam et al., 2017). Resveratrol treatment reduced α-syn oligomers in S1/S2 transfected human H4 neuroglioma cells by activating peroxisome proliferator-activated receptor γ (PPARγ), which regulates energy metabolism and mitochondrial biogenesis, and plays a role in the pathogenesis of PD (Eschbach et al., 2015). At the molecular level, resveratrol downregulates α-syn expression mediating miR-214 in the MPTP-induced mouse model of PD and MPP^+^ induced neurotoxicity in neuroblastoma cells (Wang et al., 2015a).

The phytochemicals that inhibit fibrils and oligomer formation along with the ability to stabilize the α-syn oligomers or disaggregate α-syn oligomers can be potential compounds for pharmaceutical development. The *in vitro* data reveals success with many phytochemicals in ameliorating the fibrils and oligomer formation of α-syn as well as inducing degradation of α-syn and promoting autophagy. However, many polyphenolic compounds showed difficulty in crossing the blood brain barrier due to their non-lipophilic nature. Therefore, they may not attain the required concentration to exert effects in the brain (Pandareesh et al., 2015; Pogacnik et al., 2016). Several factors, such as stability, solubility in an acidic environment at gastric pH, absorption pattern, gut microflora, enterohepatic circulation, first pass metabolism, and metabolic pattern either phase I or phase II play a key role in achieving the ideal bioavailability of the phytochemicals in the brain (Scholz and Williamson, 2007). Additionally, the inconsistency between the *in vitro* concentration and *in vivo* dose in certain models encourages systematic pharmacokinetic evaluations to understand the variation between the *in vitro* and *in vivo* data.

## Experimental Techniques to Assess the α-syn Inhibitory Activity of Phytochemicals

Several biophysical and biochemical techniques used to assess the ability of phytochemicals and plant extracts in preventing α-syn oligomerization and fibrillation are represented in Table 6. These experimental techniques include surface plasmon resonance imaging (SPRi), Thioflavin-T (ThT) fluorescence, transmission electron microscopy (TEM), small angle X-ray scattering (SAXS), circular dichroism (CD) spectroscopy, fourier transform infrared spectroscopy (FTIR), nuclear magnetic resonance (NMR), and absorption spectroscopy of Congo red (CR) binding assay (Luk et al., 2007; Kostka et al., 2008; Celej et al., 2009; Yamaguchi et al., 2010; Giehm et al., 2011; da Silva et al., 2013; Aelvoet et al., 2014; Coelho-Cerqueira et al., 2014; Cheng et al., 2015; Fazili and Naeem, 2015; Takahashi et al., 2015; Pujols et al., 2017; Das et al., 2018). The biochemical and biophysical assays employed to measure the α-syn aggregation are efficient in providing a high-resolution structure of α-syn oligomers, but not free from the restrictions and misconceptions on their efficacy (Coelho-Cerqueira et al., 2014). The polyphenolic nature of phytochemicals may cause some variations in interferences in spectrophotometric and fluorescent assays used to measure α-syn formation (Coelho-Cerqueira et al., 2014). Coelho-Cerqueira et al. (2014) show the drawbacks related to the application of ThT assays to examine α-syn fibrillation as ThT reacts with disordered α-syn monomer and augments protein fibrillation *in vitro*. As a result, phytochemicals may also bias the ThT assay and ambiguous results may be interpreted from the application of ThT based real-time assays in the screening of anti-fibrillogenic compounds. Therefore, a battery of techniques is recommended to support or confirm the anti-aggregatory and anti-fibrillogenic activity in side-stepping the possible artifacts associated with the measure of ThT fluorescence (Coelho-Cerqueira et al., 2014).

**Table 6:** The bioanalytical techniques employed to determine α-synuclein oligomerization, fibrillation, and cytotoxicity.

**Biochemical/biophysical techniques**	**Events monitored in the system**	**References**
Fluorescence polarization technique	α-syn aggregation	Luk et al., 2007
Scanning for intensely fluorescent targets and atomic force microscopy	α-syn oligomers	Kostka et al., 2008
High performance liquid chromatography (HPLC), Circular dichroism (CD), Fourier transform infrared spectroscopy (FTIR), Size exclusion HPLC, small-angle X-ray scattering, and atomic force microscopy (AFM)	HPLC (stability), Fourier transform infrared spectroscopy and atomic force microscopy (oligomer stabilization and fibrillation), CD (structural assessment)	Hong et al., 2008
Extrinsic multiple-emission probe 4′-(diethylamino)-3-hydroxyflavone spectroscopy	Amyloid fibril formed by mutant α-syn	Celej et al., 2009
Peptide mapping, Mass spectrometric and Ultra-high-field Nuclear Magnetic Resonance analysis	α-syn dimerization and inhibitor binding	Yamaguchi et al., 2010
Fluorescence spectroscopy, Thioflavin T (Thio T) assay and Transmission electron microscopy (TEM)	α-syn fibrillation and preformed α-syn	Ono and Yamada, 2006
Thio T assay, Light scattering measurement, size-exclusion HPLC, AFM	α-syn aggregation	Zhu et al., 2013
Membrane potential-sensitive bis-oxonol fluorescent dye, DiBAC4(3) bio-sensing system	Cytotoxicity of C-terminal truncated α-syn 119 (α-syn119)	Kim et al., 2013
Thio T assay, AFM, Nuclear magnetic resonance, Vesicle leakage assay	Fibril disassembling (Thio-T assay)	da Silva et al., 2013
Lipid vesicle permeabilisation assay	Membrane damage by α-syn aggregates	Caruana et al., 2012
Confocal single-molecule fluorescence spectroscopy	α-syn oligomer formation	Caruana et al., 2011
Circular dichroism spectroscopy, Transmission electron microscopy, Atomic force microscopy, and Nuclear magnetic resonance analysis and Electrophysiological assays	α-syn oligomerization, NMR (binding to the N-terminal of α-syn)	Takahashi et al., 2015
Electrochemical and localized surface plasmon resonance (LSPR), Cyclic and differential pulse voltammetry using redox probe [Fe(CN)6], Thio T assay, Surface plasmon resonance imaging, Transmission electron microscopy	α-syn oligomers by electrochemical and LSPR and Voltametry to detect binding of inhibitors to α-syn	Cheng et al., 2015
Split firefly luciferase complementation assay with bioluminescence imaging	Visualizes oligomerization of α-syn in cell culture, striatum and SNc	Aelvoet et al., 2014
ThT assays, Circular dichroism, Turbidity, and Rayleigh scattering measurements, Atomic force microscopy and Transmission electron microscopy	α-syn fibril formation	Fazili and Naeem, 2015
CCK-8 staining on MPP(+)-induced SH-SYSY cells and Transmission electron microscopy, AO staining and western blotting in cells	Survival rate (CCK-8), autophagy (TEM), AO staining (lysosome), western (α-syn)	Wang et al., 2014

The knowledge of the stage, form, nature, and propagation of α-syn that play a key role in enhanced toxicity in PD highlights the multitude of techniques needed to gauge α-syn disposition in experimental situations (Giehm et al., 2011; Pujols et al., 2017). Recently, Moree et al. (2015) developed a novel assay to recognize compounds that control the conformation of monomeric α-syn in a direct manner to reduce the encounters associated with conventional small molecule screening of α-syn. This novel assay may aid in understanding the role of α-syn oligomers in PD and opens new avenues to evaluate α-syn-based potential neuroprotective agents.

## Experimental Models for Screening Agents Targeting α-syn Toxicity

Apart from biophysical and biochemical assays, numerous models including cell lines *(in vitro)* and animal models (*in vivo*) have been developed to study the role of α-syn in the etiology and disease modification of PD and evaluate test compounds against α-syn (Skibinski and Finkbeiner, 2011; Javed et al., 2016; Visanji et al., 2016; Ko and Bezard, 2017; Lázaro et al., 2017). The experimental models showing α-syn fibrillation, oligomerization, and neurotoxicity are summarized in Table 7. The toxicant models and mutation-based *in vivo* models are popularly used to mimic sporadic and familial PD, respectively (Javed et al., 2016). The experimental models often present issues and challenges in separating the complexities of cellular and molecular mechanisms and are infeasible for high-throughput screening and other drug development stages, such as dose-response toxicology studies. Cell-based models including stem cells and primary neurons with features of dopaminergic neurons give important insights into the cellular mechanisms of PD for drug discovery (Lázaro et al., 2017). This enables the recognition of agents targeting α-syn and their molecular mechanisms. Some of the prominent human cell models for PD drug screening are SK-N-SH, SHSY5Y, and SK-N-MC (Skibinski and Finkbeiner, 2011; Lázaro et al., 2017). The development of α-syn-based experimental models of sporadic or familial PD that show progressive forms of the disease will elucidate the mechanisms of neurodegeneration and aid in the identification of phytochemicals modulating α-syn. The uptake of recombinant α-syn from the culture medium has been reported in many cellular models (Reyes et al., 2015). Recently, Reyes et al. (2015) established a culture system that is a physiologically appropriate assay for the characterization of genetic modifiers or small molecules which prevent cell-to-cell transfer or propagation of α-syn.

**Table 7:** The experimental models used to evaluate plant extracts and phytochemicals against neurotoxicity mediating α-synuclein oligomerization, and fibrillation.

**Experimental models**	**α-synuclein based pathogenesis**	**References**
MPTP-intoxicated monkeys showing PD features	Accumulation α-syn oligomers in the striatum	Chen et al., 2015b
MPP(+)-induced toxicity in SH-SY5Y cells	Increased α-syn level and expression	Wang et al., 2014
Rotenone-induced neurotoxicity in cell lines	Increased α-syn aggregation and synphilin-1 deposits	Kabiraj et al., 2014
Rotenone-induced neurotoxicity in SH-SY5Y cells	Increased α-syn level and expression	Deng et al., 2013
Lipid vesicles and SH-SY5Y cells	Formation of Aβ42, α-syn and tau aggregate complexes	Camilleri et al., 2013
Cytotoxicity in catecholaminergic PC12 cells	Increased α-syn level and expression	Teraoka et al., 2012
MPTP/MPP+-induced neurotoxicity in PC12 cells	Increased α-syn level and expression	Patel et al., 2012
Dopamine-induction in SH-SY5Y cells	Increased α-syn expression	Ham et al., 2013
Rotenone-neurotoxicity in SH-SY5Y and PC12 cells	Enhanced degradation of α-syn	Wu et al., 2011
Transgenic Drosophila expressing human α-syn	Increased α-syn l expression	Long et al., 2009
Yeast-based model expressing α-syn	Increased α-syn fibrillation-induced neurotoxicity	Griffioen et al., 2006
MPTP and 6-OHDA-induced PD in rodents	Increased of α-syn expression in the SNpc	Mandel et al., 2004

## Concluding Remarks and Future Prospects

This comprehensive review presents an overview of the plant extracts and phytochemicals specifically targeting α-syn oligomerization, fibrillation, and aggregation in different models of PD and their underlying mechanisms. It also discusses the experimental techniques and models used to evaluate the plant extracts and phytochemicals. The literature review suggests that many phytochemicals are promising in targeting α-syn in the *in vitro* studies; however, the actions observed *in vitro* need to be reconfirmed *in vivo*. Indeed, the screening of phytochemicals or plant extracts in cell lines often lacks clinical applicability due to physiological, biochemical, and pharmacological relevancy. The available literature from a convincing number of *in vitro* studies and few *in vivo* studies demonstrates that phytochemicals, such as baicalein, curcumin, resveratrol, and epigallocatechin gallate have promising therapeutic potential in inhibiting α-syn oligomerization, fibrillation, aggregation, and accumulation. All these promising compounds should be studied in the *in vivo* studies to proceed further for clinical studies and thereon.

The process of α-syn oligomerization and fibrillation were well-recognized but the triggers that induce α-syn aggregation are not yet well-established. Thus, for a fair translational disease modifying approach, evaluation of phytochemicals in animal models involving α-syn aggregation and mimicking the progressive nature of PD pathogenesis is desired as a proof of concept. Although the available preclinical studies are encouraging, they are markedly speculative for clinical success. The issues, such as bioavailability, stability, metabolism, as well as long-term safety and toxicity, should be resolved before pharmaceutical development and further testing in humans. Based on the available preclinical studies, it can be concluded that these phytochemicals could possibly be novel drug candidates for neurodegenerative diseases, such as PD.

## Author Contributions

All the authors provided important intellectual content, reviewed the content and approved the final version of this manuscript. AA and SO conceptualized the idea for this review. MFNM, SA, HJ, and SO performed the literature search. HJ and SO wrote the first draft of the manuscript. SO and AA thoroughly revised and edited the manuscript. BS drew the chemical structures.

### Conflict of Interest Statement

The authors declare that the research was conducted in the absence of any commercial or financial relationships that could be construed as a potential conflict of interest.
